# Computationally designed hyperactive Cas9 enzymes

**DOI:** 10.1038/s41467-022-30598-9

**Published:** 2022-05-31

**Authors:** Pascal D. Vos, Giulia Rossetti, Jessica L. Mantegna, Stefan J. Siira, Andrianto P. Gandadireja, Mitchell Bruce, Samuel A. Raven, Olga Khersonsky, Sarel J. Fleishman, Aleksandra Filipovska, Oliver Rackham

**Affiliations:** 1 Curtin Medical School, Curtin University, Bentley, WA Australia; 2 Curtin Health Innovation Research Institute, Curtin University, Bentley, WA Australia; 3 Harry Perkins Institute of Medical Research, QEII Medical Centre, Nedlands, WA Australia; 4 ARC Centre of Excellence in Synthetic Biology, QEII Medical Centre, Nedlands, WA Australia; 5 Centre for Medical Research, The University of Western Australia, Nedlands, WA Australia; 6 School of Molecular Sciences, The University of Western Australia, Crawley, WA Australia; 7 Telethon Kids Institute, Northern Entrance, Perth Children’s Hospital, Nedlands, WA Australia; 8 Department of Biomolecular Sciences, Weizmann Institute of Science, Rehovot, Israel

**Keywords:** Protein design, CRISPR-Cas9 genome editing

## Abstract

The ability to alter the genomes of living cells is key to understanding how genes influence the functions of organisms and will be critical to modify living systems for useful purposes. However, this promise has long been limited by the technical challenges involved in genetic engineering. Recent advances in gene editing have bypassed some of these challenges but they are still far from ideal. Here we use FuncLib to computationally design Cas9 enzymes with substantially higher donor-independent editing activities. We use genetic circuits linked to cell survival in yeast to quantify Cas9 activity and discover synergistic interactions between engineered regions. These hyperactive Cas9 variants function efficiently in mammalian cells and introduce larger and more diverse pools of insertions and deletions into targeted genomic regions, providing tools to enhance and expand the possible applications of CRISPR-based gene editing.

## Abstract (web summary)

The ability to alter the genomes of living cells is key to understanding how genes influence the functions of organisms and will be critical to modify living systems for useful purposes. Here, the authors use computational design to discover Cas9 enzymes with increased activity.

## Introduction

The myriad applications of Cas9 have significantly accelerated biological research^1–4^, however, a number of unsolved issues still remain. Perhaps the most surprising limitation to the CRISPR-Cas system is that Cas9 has been found to be particularly inefficient in its cleavage activity. Although the majority of DNA substrates can be cleaved in seconds^5^, the cutting half-life and catalytic lifetime for *Streptococcus pyogenes* Cas9 (Cas9) are both ~6 h^2,6,7^, which compares unfavorably with other nucleases, such as restriction enzymes. The DNA cleavage rate therefore becomes limited by the time required for Cas9 to dissociate from its DNA substrate and resample the population of target sites in a cell. In fact, kinetic studies have revealed that Cas9 is effectively a single turnover nuclease^5,8–10^. However, the precise in vivo kinetics of Cas9 cleavage can be target strand and locus-dependent, given that other cellular processes, such as transcription, are thought to promote post-cleavage dissociation and thus turnover in a context-dependent manner^11^. Interestingly, it is often seen that mutations designed to lower Cas9 off-target activity result in a decreased affinity for its target sequence and reduced mutagenesis rate, thus exacerbating the low efficiency problem^12^. For many applications in cells and in vitro Cas9 enzymes with higher catalytic efficiency would be particularly beneficial.

**Fig. 1:**
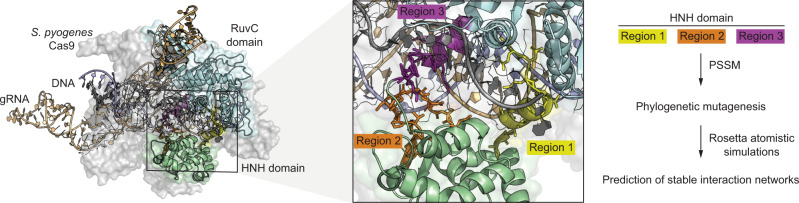
Computational design of hyperactive Cas9 enzymes. The crystal structure of the *Streptococcus pyogenes* Cas9 poised to cleave DNA (PDB accession code 5F9R)^21^ with the HNH and RuvC nuclease domains highlighted in green and blue, respectively. The inset shows the three regions of the HNH domain targeted for computational mutagenesis and the conceptual approach is shown schematically.

In this study, we engineered the HNH-like nuclease domain of Cas9 (Fig. 1) to substantially increase the frequency of donor-independent gene editing through computational design. Recently, we developed a computational design approach, called FuncLib, that mutates positions within the active sites of enzymes to increase their catalytic rate or specificity^13^. The FuncLib approach combines phylogenetic analysis of sequence homologs with Rosetta atomistic design calculations. The resulting designs each exhibit several mutations in the enzyme active site that are predicted to form low-energy constellations, while conserving the key catalytic residues in their active conformation. Furthermore, the designs are highly diverse, exhibiting several mutations relative to one another and the wild-type enzyme. We and others have demonstrated several cases in which FuncLib yielded large gains in efficiency or specificity increases and that unlike in vitro evolution, it does not require screening more than several dozen designs^14^. Crucially, FuncLib has been successfully applied to design enzymes in their *apo* states, suggesting that it can be applied to cases, like Cas9, which are dynamic and in which not all the conformations that are relevant for the catalytic cycle have been characterized structurally.

## Results

### Computational design and testing of Cas9 efficacy using a synthetic genetic circuit in yeast

**Fig. 2:**
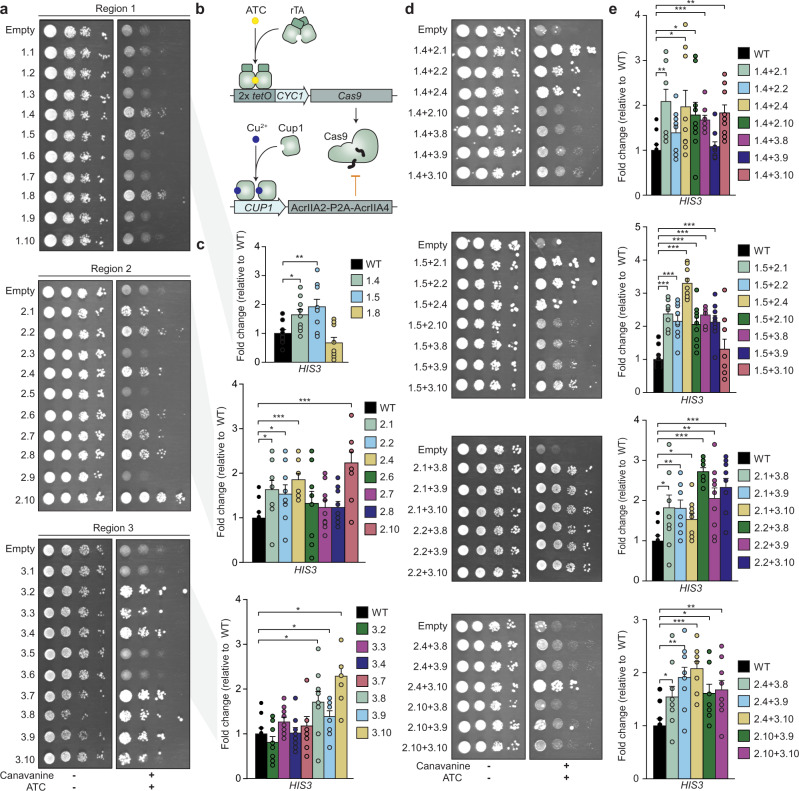
Qualitative and quantitative screening of hyperactive Cas9 enzymes in *Saccharomyces cerevisiae*. **a** Yeast survival assays examining the functionality of individual Cas9 designs targeting *CAN1* via survival on canavanine. **b** Schematic representation of the quantitative Cas9 inhibitor-modulated genetic circuit. **c** Quantitation of engineered Cas9 activity via yeast survival with the Cas9 inhibitor system and a gRNA targeting *HIS3*. **d** Survival assays to determine the functionality of engineered Cas9 enzymes combining multiple designed regions. **e** Quantification of the activities of combined Cas9 designs. Error bars: s.e.m. of *n* = 9 biologically independent replicates. A standard Student’s *t*-test with a two-tailed distribution and unequal variance assumed between samples was used to calculate the significance. p-values: * *p* < 0.05, ** *p* < 0.01, ***p < 0.001. Specific p-values for panel **c** design vs WT: 1.4 *p* = 0.01, 1.5 *p* = 0.008, 2.1 *p* =0.02, 2.2 *p* = 0.05, 2.4 *p* = 0.0003, 2.10 *p* = 0.0007, 3.8 *p* = 0.02, 3.9 *p* = 0.05 and 3.10 *p* =0.0006 (Student’s *t*-test). Specific p-values for panel **d** design vs WT: 1.4 + 2.1 *p* = 0.004, 1.4 + 2.4 *p* = 0.03, 1.4 + 2.10 *p* = 0.03, 1.4 + 3.8 *p* = 0.001, 1.4 + 3.10 *p* = 0.002, 1.5 + 2.1 *p* = 1.4e-06, 1.5 + 2.2 *p* = 8.2e-05, 1.5 + 2.4 *p* = 4.9e-09, 1.5 + 2.10 *p* = 6.2e-04, 1.5 + 3.8 *p* = 6.7e-07, 1.5 + 3.9 *p* = 1.4e-04, 2.1 + 3.8 0.04, 2.1 + 3.9 *p* = 0.005, 2.1 + 3.10 *p* = 0.02, 2.2 + 3.8 *p* = 2.5e-08, 2.2 + 3.9 *p* = 0.004, 2.2 + 3.10 *p* = 0.0002, 2.4 + 3.8 *p* = 0.04, 2.4 + 3.9 *p* = 0.001, 2.4 + 3.10 *p* = 3.2e-05, 2.10 + 3.9 *p* = 0.01 and 2.10 + 3.10 *p* = 0.009 (Student’s *t*-test). Source data are provided as a Source Data file.

We focused our engineering efforts on the HNH nuclease domain as it orchestrates Cas9 cleavage, moving between multiple different conformations during the catalytic cycle, and also regulates cleavage by Cas9’s other active site, the RuvC-like nuclease domain^5,10,15^. Based on several crystal structures, three separate regions in the HNH domain-containing amino acids 765–780, 838–853, and 911–924 (Fig. 1) were chosen for in silico design using FuncLib. These three regions were chosen as they are either in contact with the target DNA or are required to position active site residues for enzymatic cleavage (Supplementary Fig. 1a–d). For each region, the 10 lowest energy designs according to FuncLib were assessed for enzymatic activity by coupling Cas9 cleavage with cell survival in yeast (Supplementary Fig. 1e). We designed a system consisting of a tetracycline-inducible Cas9 expression plasmid and a constitutive gRNA plasmid (Supplementary Fig. 1f). Specific gRNAs were designed to knock out the open reading frames of the auxotrophic genes *ADE2* and *HIS3*. Loss of function of these genes prevents the growth of yeast on media lacking either adenine or histidine, respectively. In addition, we also used a gRNA targeting *CAN1*, encoding an arginine permease whose loss enables survival in the presence of canavanine. Cas9 designs were tested using each gRNA separately to determine if the mutations were compatible with Cas9 function. Mutation of *ADE2* and *HIS3* reduced growth in the absence of adenine or histidine, respectively, while targeting *CAN1* rescued growth on media containing canavanine (Supplementary Fig. 1g). Three designs for region 1 (1.4–1.5, 1.8) were found to be catalytically active, while regions 2 and 3 each produced 7 active designs (2.1–2.2, 2.4, 2.6–2.8, 2.10, 3.2–3.4, and 3.7–3.10) (Fig. 2a).

While our system was highly effective in establishing the functionality of wild-type Cas9 (Supplementary Fig. 1g) and our Cas9 designs (Fig. 2a, d) using all three gRNAs, we found that the targeted yeast genes were rapidly mutated upon transformation, complicating a detailed comparison of catalytic activities. To eliminate this confounding variable, we introduced two known Cas9 inhibitors, AcrIIA2 and AcrIIA4, which were shown to bind in distinct modes to the Cas9-gRNA complex^16^. Notably, it has been found that mutations in Cas9 that eliminate the inhibitory effect of AcrIIA2 have no effect on inhibition of Cas9 activity by AcrIIA4 and vice versa^16^. AcrIIA2 and AcrIIA4 were fused by a self-cleaving peptide (P2A) and their expression was controlled with a copper-inducible promoter (Fig. 2b, Supplementary Fig. 1h). Using this method, we were able to inhibit preemptive Cas9 activity with 100 mM copper sulfate, while allowing efficient induction of Cas9 in the absence of copper (Supplementary Figs. 1i and 2). This enabled quantification of the in vivo activity of designer Cas9 enzymes targeting *ADE2* and *HIS3* in comparison to wild-type Cas9 via cell survival mediated through loss of either *ADE2* or *HIS3* (Fig. 2b and Supplementary Fig. 1j). We observed significant fold changes in activity for 9 designs ranging from 1.4-fold for design 3.9 to 2.3-fold for design 3.10 using the *HIS3* gRNA compared to wild-type Cas9 (Fig. 2c). Interestingly, only 2 out of 17 designs (1.4 and 2.4) were found to have a significant decrease in survival and a significant fold change in activity for both the *ADE2* and *HIS3* gRNAs (Fig. 2c and Supplementary Fig. 3a–g). Taken together, these data demonstrate a strength of the FuncLib approach compared, for instance, to in vitro evolution, since nearly a third of the HNH nuclease-domain designs were significantly more active than their wild-type counterpart, allowing us to choose the most active ones through small-scale experimental testing.

Each of the designs in the three different regions were computed independently of one another, and as such might not be compatible with each other. We hypothesized, however, that there could be a potential to further increase the enzymatic activity by combining designs from different regions. We made designs with all possible combinations of the mutated regions that had a significant increase in activity and found that all combinations, with the exception of design 2.10 + 3.8, retained their enzymatic activity (Fig. 2d). Furthermore, most combinations were found to have a significant increase in activity when compared to wild-type Cas9 for both the *ADE2* and *HIS3* gRNAs (Fig. 2e and Supplementary Fig. 4a–i). However, in order to establish that the combinations result in a synergistic increase in activity, we compared the activity of each combination relative to their single mutant counterparts (e.g. design 1.4 + 2.1 compared to both designs 1.4 and 2.1) (Supplementary Fig. 4j). Testing using the *HIS3* gRNA showed that most combined designs were found to have either a neutral effect (~1-fold change, *p* > 0.05) or a positive fold change (fold change >1.0, *p* < 0.05). Only designs 1.4 + 3.9 and 1.5 + 3.10 were found to have negative epistasis (fold change <1.0, *p* < 0.05) for the *HIS3* gRNA and none demonstrated a significant improvement in activity for the *ADE2* gRNA compared to the individual mutants (Supplementary Fig. 3j).

### Hyperactive Cas9 functionality in mammalian cells

**Fig. 3:**
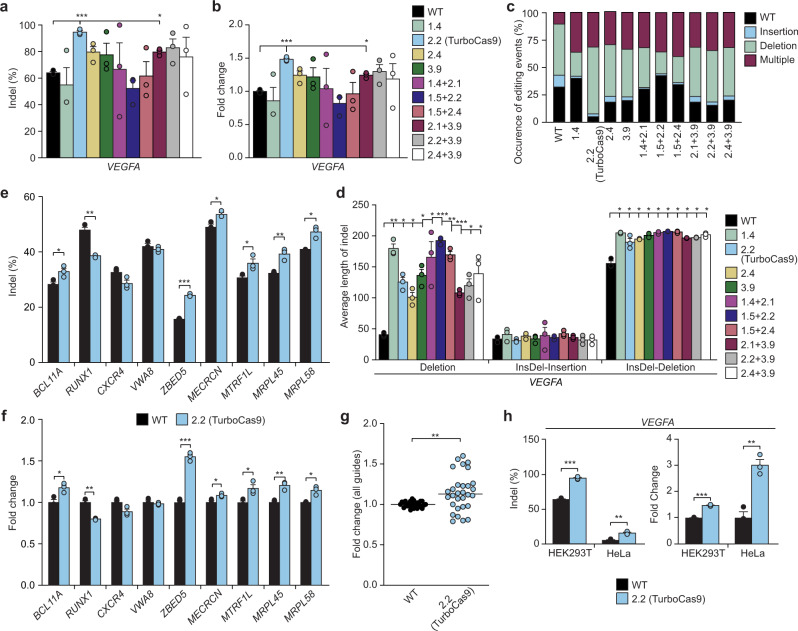
Hyperactive Cas9 enzymes effectively generate large and complex mutations in mammalian cells. **a** Percentage of indels introduced into the *VEGFA* gene by engineered Cas9 enzymes in HEK293T cells. **b** Fold change in Cas9 activity of selected mutants relative to wild-type Cas9. **c** Engineered Cas9 enzymes produce more complex, multiply edited mutations. **d** Engineered Cas9 enzymes introduce significantly larger deletions. **e** Percentage of indels introduced into *BCL11A*, *RUNX1*, *CXCR4*, *VWA8*, *ZBED5*, *MTRF1L*, *MECRCN*, *MRPL45* and *MRPL58* genes. **f** Fold change in designed Cas9 activity relative to wild-type Cas9. **g** Fold change distribution for individual samples for all 10 gRNAs. ANOVA testing identified that the increase in the activity of TurboCas9 was significant when considered across all mammalian gRNAs, with a significant difference between wild-type and TurboCas9 overall (*p*-value <0.0001) as well as for each guide (*p*-value <0.0001). **h** The type of cell line used has a significant effect on the efficiency of Cas9 and TurboCas9 between HEK293T and HeLa cells, displayed in percentage edited and fold change relative to wild-type Cas9 for the *VEGFA* gene. Error bars: s.e.m. for *n* = 3 biologically independent replicates. An FDR-adjusted Student’s *t*-test with a two-tailed distribution and unequal variance assumed between samples was used to calculate the significance. *p*-values: *p < 0.05, **p < 0.01, ***p < 0.001. Specific *p*-values for panels **a**, **b** for design vs WT: 2.2 *p* = 0.0006 and 2.1 + 3.9 *p* = 0.01 (FDR-adjusted Student’s *t*-test). Specific *p*-values for **d** for design vs WT for Deletions: 1.4 *p* = 0.004, 2.2 *p* = 0.01, 2.4 *p* = 0.02, 3.9 *p* = 0.01, 1.4 + 2.1 *p* = 0.04, 1.5 + 2.2 *p* = 0.0003, 1.5 + 2.4 *p* = 0.002, 2.1 + 3.9 *p* = 0.0005, 2.2 + 3.9 *p* = 0.02, 2.4 + 3.9 *p* = 0.05; for InsDel-Deletions: 1.4 *p* = 0.01, 2.2 *p* = 0.01, 2.4 *p* = 0.01, 3.9 *p* = 0.01, 1.4 + 2.1 *p* = 0.01, 1.5 + 2.2 *p* = 0.01, 1.5 + 2.4 *p* = 0.01, 2.1 + 3.9 *p* = 0.01, 2.2 + 3.9 *p* = 0.01 and 2.4 + 3.9 p=0.01 (FDR-adjusted Student’s *t*-test). Specific *p*-values for **e**: *BCL11A* *p* = 0.03, *RUNX1* *p* = 0.01, *ZBED5* *p* = 0.0003, *MTRF1L* p=0.05, *MECRCN* *p* = 0.05, *MRPL45* *p* = 0.009 and *MRPL58* *p* = 0.04 (FDR-adjusted Student’s *t*-test). Specific *p*-values for **f** for design 2.2 (TurboCas9) vs wild-type Cas9: *BCL11A* p=0.03, *RUNX1* p=0.01, *ZBED5* *p* = 0.0003, *MTRF1L* *p* = 0.05, *MECRCN* *p* = 0.05, *MRPL45* *p* = 0.009 and *MRPL58* *p* = 0.04 (FDR-adjusted Student’s *t*-test). For panel **g** *p* = 0.001 from a Student’s *t*-test for TurboCas9 vs wild-type Cas9 for all guides. For **h** the specific *p*-values for TurboCas9 vs wild-type Cas9 for HEK293T *p* = 0.0006 and HeLa *p* = 0.004 (FDR-adjusted Student’s *t*-test).

We selected the 10 most active Cas9 designs for testing in mammalian systems. These designs were codon optimized for mammalian-cell expression. We used a well-characterized *VEGFA* gRNA, with known off target cleavage sites, and determined donor-independent editing efficiencies in human HEK293T cells by next-generation sequencing of targeted DNA amplicons. Several designs showed a significant increase in the number of mutated sequences at the *VEGFA* locus, particularly designs 2.2 and 2.1 + 3.9, with 95% and 79%, respectively, of *VEGFA* alleles edited (Fig. 3a and Supplementary Fig. 5a), whereas wild-type Cas9 mutated 64% of *VEGFA* alleles. This result represents a 1.5-fold improvement in editing for design 2.2, which we dub TurboCas9, and a 1.2-fold increase for design 2.1 + 3.9 (Fig. 3b). Several other Cas9 designs were trending towards improved editing, but these were not statistically significant, while the others remained as active as the wild-type Cas9.

We developed a computational pipeline to classify donor-independent editing into three broad categories: single events of either a deletion or insertion, and combined events in which insertion and deletion or multiples thereof occurred within the same allele (“Multiple”)(Fig. 3c). Wild-type Cas9-mediated editing resulted predominantly in single deletion and insertion events; however, combined events were comparatively sparse (Fig. 3c). Single deletion events occurred at a similar rate for the Cas9 designs and were not significantly different from wild-type Cas9. Our designs had an approximately two-fold decrease in the number of insertions (Fig. 3c), although the insertion lengths were similar (Supplementary Fig. 5b). Still, the designs caused a three-fold or more increase in the number of multiply edited alleles (Fig. 3c). The accumulation of indels has been shown to be dependent on the rate at which cleaved DNA is produced and made available to the cellular repair machinery by editing enzymes^6^, indicating that the designed mutations successfully increased the activity of Cas9. Furthermore, in addition to the number of resulting mutations, every one of the engineered Cas9 enzymes induced significantly larger deletions (Fig. 3d). Increases in the sizes of the deletions for single events ranged from ~two-fold for design 2.4 to well over four-fold for design 1.5 + 2.2.

TurboCas9 cleavage of the *VEGFA* gene caused a large number of deletions that nearly consumed the entire amplicon (Supplementary Fig. 6). To examine whether the size of the target region for amplicon sequencing might obscure even larger deletions we amplified a 4.2 kb region of the *VEGFA* locus surrounding the gRNA target site. Agarose gel electrophoresis revealed a minor proportion of smaller PCR products in *VEGFA* alleles targeted by wild-type Cas9 and this was amplified in samples treated with TurboCas9 (Supplementary Fig. 5k), indicating that there are several large deletions that would not have been detected in standard amplicon sequencing. Long amplicons were also subjected to tagmentation followed by deep sequencing to catalog the types of large deletions present. These data revealed a preponderance of large deletions ranging up to 3.9 kb in size that was more frequent in cells expressing TurboCas9 (Supplementary Fig. 5l, m). Taken together, we conclude that the designs significantly increase Cas9 activity as well as improving the enzyme’s ability to generate indels that create a knockout or delete larger parts of target genes.

Next we examined the generality of these observations using additional diverse gRNAs. These included a therapeutic gRNA targeting the *BCL11A* gene, a selection of gRNAs that were verified in our laboratory previously (*ZBED5*, *MECRCN*, *MRPL45*, *MRPL58*, *MTRF1L*, *VWA8*), and an additional two from the literature (*CXCR4* and *RUNX1*). We found that TurboCas9 significantly enhanced donor-independent editing with six of these gRNAs (*ZBED5*, *MECRCN*, *MRPL45*, *MRPL58* and *MTRF1L*), had no effect on editing by two gRNAs (*VWA8* and *CXCR4*), and decreased editing for one gRNA (*RUNX1*) (Fig. 3e, f). Therefore, for the majority of gRNAs TurboCas9 enables more effective gene disruption (Fig. 3g). Given the increased activity of TurboCas9 that was observed in HEK293T cells, we examined donor-independent editing of our most active mutant TurboCas9 in HeLa, another commonly used cell line (Fig. 3h). Interestingly, even though HeLa cells exhibit a lower level of donor-independent editing using the *VEGFA* gRNA than HEK293T cells, likely due to the fact that HEK293T cells are partially deficient in DNA mismatch repair^17^, we observed that TurboCas9 was threefold more active than wild-type Cas9. This indicates that TurboCas9 might be particularly useful in systems where gene editing is typically less efficient.

Increasing Cas9 activity would result in a requirement for an increased number of repair events and thus potentially increase the complexity of DNA repair outcomes at these sites. To examine the nature of the induced mutations in more detail, we mapped the exact locations and lengths of mutations for the *VEGFA* donor-independent experiments (Supplementary Fig. 6) and categorized indel events based on their respective CIGAR (concise idiosyncratic gapped alignment report) complexity level (Supplementary Fig. 5c), where the higher the CIGAR complexity (CC) levels comprise deletions and insertions occurring simultaneously in more complex combinations. We observed that the number of reads categorized in these higher CIGAR complexity levels in our designs was significantly increased relative to wild-type Cas9 (Supplementary Fig. 5d). All mutants were found to have at least a twofold increase in the number of reads in CC4, TurboCas9 had an approximately threefold increase in the number of reads present in CC6 and CC7, and TurboCas9, design 1.5 + 2.4 and 2.1 + 3.9 were found to have a significant increase of alleles in CC7 (Supplementary Fig. 5d), which include multiple deletions and multiple insertions within a single allele. We did not find a significant change in the occurrence of frameshifts as a result of all editing events combined (Supplementary Fig. 5e), although the larger single deletion events induced by the designs resulted in significantly more frameshifts in 8 out of 10 designs (Supplementary Fig. 5f). Several of our designs are either trending towards or have significantly increased activity relative to wild-type Cas9; however, all designs increased the number of complex editing events in comparison to wild-type Cas9.

**Fig. 4:**
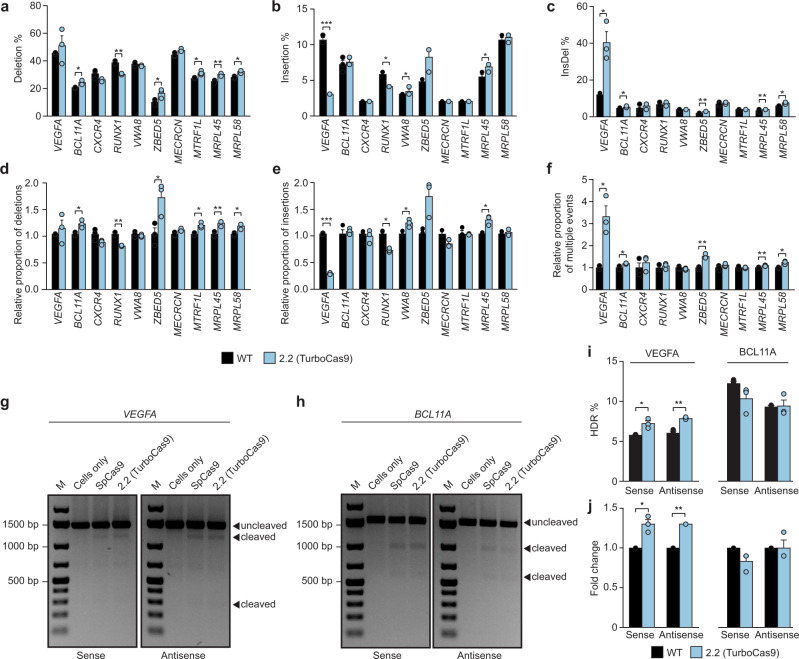
An engineered Cas9 variant is highly effective at generating deletions and multiply edited complex events. **a**–**c** Occurrence of editing events classified as **a** deletions **b** insertions **c** multiply edited complex mutations. **d**–**f** Relative occurrence of **d** deletions **e** insertions **f** multiply edited complex events. **g** PstI digestion of *VEGFA* HDR samples. M denotes the molecular weight marker. **h** PstI digestion of *BCL11A* HDR samples. **i** Occurrence of HDR in percentage. **j** Fold change of HDR. Error bars: s.e.m. for *n* = 3 biologically independent replicates. A standard Student’s *t*-test with a two-tailed distribution and unequal variance assumed between samples was used to calculate the significance. *p*-values: *p < 0.05, **p < 0.01, ***p < 0.001. Specific *p*-values for **a** for TurboCas9 vs wild-type Cas9: *BCL11A* *p* = 0.02, *RUNX1* *p* = 0.005, *ZBED5* *p* = 0.04, *MTRF1L* *p* = 0.02, *MRPL45* *p *= 0.008 and *MRPL58* *p* = 0.02; for **b**: *VEGFA* *p *= 7.0e-07, *RUNX1* *p* = 0.02, *VWA8* *p* = 0.02 and *MRPL45* *p* = 0.02;  for **c**: *VEGFA* *p* = 0.04, *BCL11A* *p* = 0.04, *ZBED5* *p* = 0.003, *MRPL45* *p* = 0.009 and *MRPL58* *p* = 0.02 (FDR-adjusted Student’s *t*-test). Specific p-values for **d** for TurboCas9 vs wild-type Cas9: *BCL11A* *p* = 0.02, *RUNX1* *p* = 0.005, *ZBED5* *p* = 0.04, *MTRF1L* *p* = 0.02, *MRPL45 p* = 0.008 and *MRPL58 p* = 0.02; for **e**: *VEGFA p* = 7.0e-07, *RUNX1 p *= 0.02, *VWA8 p* = 0.02 and *MRPL45 p* = 0.02; for **f**: *VEGFA* *p* = 0.04, *BCL11A* *p* = 0.04, *ZBED5* *p* = 0.003, *MRPL45* *p* = 0.009 and *MRPL58* *p* = 0.02 (FDR-adjusted Student’s *t*-test). Specific *p*-values for **i**: sense oligonucleotide *p* = 0.05 and antisense oligonucleotide *p* = 0.01. Specific *p*-values for **j**: sense oligonucleotide *p* = 0.05 and antisense oligonucleotide *p* = 0.01 (FDR-adjusted Student’s *t*-test). Source data are provided as a Source Data file.

For most of the other gRNAs tested the average length of all different indel types was not significantly changed (Supplementary Fig. 5g-j). However, we found that for *VWA8*, although its level of mutation was not changed significantly, it demonstrated a significant increase in the length of insertions as single events as well as in the context of multiply edited events. Furthermore, we found that *ZBED5* and *MRPL58* genes targeted by TurboCas9 had a significant increase in the average length of deletions. While *MRPL58* and *RUNX1* exhibited a significant increase in the average length of deletions in the context of multiply edited alleles. The frequency at which deletions or multiply edited events occur was increased for TurboCas9 with six out of 10 gRNAs and five out of 10 gRNAs, respectively (Fig. 4a-f). In contrast, insertions were only significantly increased for two out of 10 gRNAs. These data are in line with a trend we observed for all of our Cas9 designs as all show a reduced level of single insertion events, whilst providing a significant increase in deletions and multiply edited events.

### Efficacy of TurboCas9 in homology directed repair

Although harnessing non-homologous end joining to mutate target genes is currently the most common application of CRISPR, using Cas9 to stimulate homology-directed repair (HDR) to make more substantial and scarless genome modifications is highly desirable. To examine whether it is possible to perform HDR using TurboCas9, we targeted *BCL11A* and *VEGFA* loci with homologous single-stranded oligonucleotide templates that carry several mutations within the gRNA to introduce a PstI recognition site. PstI digestion of amplicons generated from these samples showed that efficient levels of HDR were achieved for both wild-type Cas9 as well as TurboCas9 (Fig. 4g–j). We sequenced short amplicons flanking the HDR site and found that levels of HDR were similar for oligonucleotides corresponding to both the sense and antisense strands for both target genes (Fig. 4i–j). Interestingly, although wild-type Cas9 and TurboCas9 stimulated similar levels of HDR at the *BCL11A* locus, TurboCas9 enabled significantly more HDR events at the *VEGFA* target site. These data indicate that TurboCas9 is compatible with HDR approaches and in some cases may enable more efficient HDR.

### Fidelity of hyperactive Cas9 enzymes in mammalian cells

**Fig. 5:**
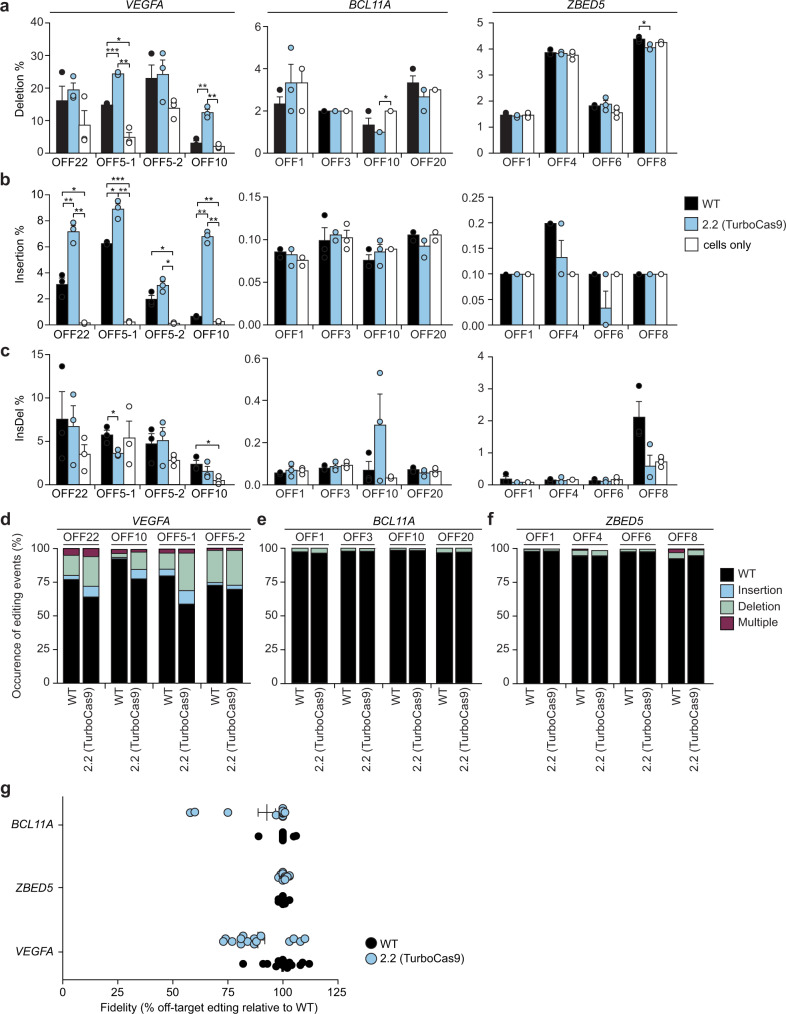
Off-target DNA editing in mammalian cells. **a**–**c** Percentage of reads for *VEGFA*, *BCL11A* and *ZBED5* that have **a** deletions **b** insertions and **c** combined insertions and deletions (Insdel). **d**–**f** The occurrence of different editing events varies between Cas9 variants and off-target sites for **d** VEGFA **e** *BCL11A* and **f** *ZBED5*. **g** Overall fidelity of engineered Cas9 relative to wild-type Cas9 for *BCL11A*, *ZBED5* and *VEGFA*, calculated as off-target error rate as a proportion of wild-type error rate. Error bars: s.e.m. for *n* = 3 biologically independent replicates. An FDR-adjusted Student’s *t*-test with a two-tailed distribution and unequal variance assumed between samples was used to calculate the significance. *p*-values: *p < 0.05, **p < 0.01, ***p < 0.001. Specific *p*-values for **a** for TurboCas9 vs wild-type Cas9: *VEGFA* OFF5-1 *p* = 0.00002, OFF10 *p* = 0.002, for *ZBED5* OFF8 *p* = 0.02; cells only vs wild-type Cas9: *VEGFA* OFF 5-1 *p* = 0.02, *BCL11A* OFF8 *p* = 0.04; cells only vs TurboCas9: *VEGFA* OFF 5-1 *p* = 0.005, OFF10 *p* = 0.004, *BCL11A* OFF10 *p *= 0.02 (FDR-adjusted Student’s *t*-test). Specific *p*-values for **b** for TurboCas9 vs wild-type Cas9: *VEGFA* OFF22 *p* = 0.004, OFF5-1 *p* = 0.02, OFF10 *p* = 0.001; cells only vs wild-type Cas9: *VEGFA* OFF 5-1 *p* = 0.00002, OFF22 *p* = 0.03, OFF5-2 *p* = 0.03, OFF10 *p* = 0.002; cells only vs TurboCas9: *VEGFA* OFF 5-1 *p* = 0.002, OFF22 *p* = 0.004, OFF10 *p* = 0.001, OFF5-2 *p* = 0.01 (FDR-adjusted Student’s *t*-test). Specific *p*-values for **c** cells only vs wild-type Cas9 for *VEGFA* OFF10 *p* = 0.03; TurboCas9 vs wild-type Cas9: OFF22 *p* = 0.05 (FDR-adjusted Student’s *t*-test).

Increased fidelity has been observed to be inversely correlated with on-target activity^12^. Therefore, we examined whether Cas9 designs that increase on-target activity would exhibit a similar increase in off-target activity. We amplified the top 4 known off-target sites for the *VEGFA* gRNA, named OFF22, OFF10, OFF5-1, and OFF5-2, after editing by TurboCas9, compared to wild-type Cas9. Interestingly, we observed that the designs increased editing at two off targets but did not significantly increase editing at two other off-targets (Fig. 5a–d and Supplementary Figs. 7-9). OFF5-2 differs from the *VEGFA* gRNA by two bp with one mismatch occurring at base 18 of the seed sequence, which is typically less tolerated by Cas9 and corroborated in our data by the low levels of editing for the wild-type Cas9. The increased activity of TurboCas9 does not seem to have lessened the fidelity of Cas9 when mismatches between the seed sequence and the target occur near the PAM sequence. OFF22 has a mismatch at bp 14 of the gRNA sequence and no significant difference was observed between our designs and the wild-type Cas9. OFF10 and OFF5-1 were both found to have been edited significantly more by our designs and both have mutations in the first 10 bp of the gRNA. Unlike the on-target site, we did not observe an increase in multiply edited alleles nor a reduction in insertions for these off-target sites (Fig. 5d, Supplementary Figs. 8 and 9). Similar observations were found for the distribution of reads in the different levels of CIGAR complexity (Fig. 5d, Supplementary Fig. 7a, b). Interestingly, the previously seen increase in deletion size for both the single deletions and also deletions within multiply edited alleles for the engineered Cas9 enzymes was not observed for the off-target sites. On the contrary, for several of the off-target sites, we observed a significant decrease in deletion size. Thus, the designs significantly increase Cas9 on-target activity without a consistently negative impact on fidelity. To extend this observation, we predicted off-target sites for each of the gRNAs targeting *ZBED5* and *BCL11A* and used amplicon sequencing to compare TurboCas9 and wild-type Cas9. We observed overall low levels of editing for these off-target sites, similar to untreated cells, with no significant differences between TurboCas9 and wild-type Cas9. The average length of each of the four classified indel types also showed no significant differences (Supplementary Fig 7c, d). Taken together all the data examining the fidelity of TurboCas9 versus wild-type Cas9 showed we achieved similar levels of fidelity while significantly increasing the on-target activity (Fig. 5g).

### Structural analysis of the TurboCas9 designed mutations

**Fig. 6:**
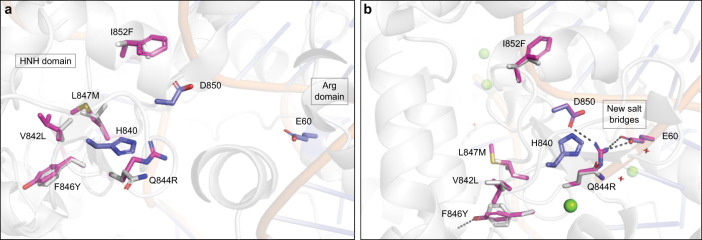
Structural impact of mutations in the TurboCas9 design. The mutated region is shown **a** on the structure used for the design (PDB accession code 5F9R) and **b** on the more recent structure with catalytically relevant H840 position and Mg^2+^ ions (PDB accession code 7S4X). The mutated residues are shown as sticks (WT identities shown in gray, designed identities in magenta), and the catalytic H840, and D850 with E60 are shown as blue sticks. Mg^2+^ ions are shown as green spheres. In **a**, mutation Q844R improves the solvation by introducing a charged residue on the protein surface, and in **b**, it forms new salt bridges with D850 and E60 (from the Arg domain) and can specifically stabilize the active form. Mutations V842L, L847M, and I852F optimize the core packing, and mutation F846Y introduces a new hydrogen bond to the protein backbone carbonyl.

To understand the structural basis for the improved efficiency observed in TurboCas9, we examined its design model. When we conducted the design calculations, the most relevant molecular structure of the Cas9 active conformation was PDB entry 5F9R. This structure, however, places the catalytic His840 distant from the DNA and does not include the Mg^2+^ ions that are crucial for catalytic activity. One of the five mutations in TurboCas9, Gln844Arg, is in close proximity to the catalytic His840 and its role in improving activity cannot be understood in the context of the molecular structure which we had available at the time (Fig. 6a and Supplementary Fig. 10a). We therefore modeled the five TurboCas9 mutations on a more recent structure (PDB entry: 7S4X)^18^, which has the His840 in a catalytically competent position as well as the crucial Mg^2+^ ions (Fig. 6b). This model shows that Arg844 may form stabilizing saltbridge interactions with Asp850 (within the HNH domain) and Glu60 (within the Arg domain). Thus, the Gln844Arg mutation is tolerated in the inactive HNH conformation where position 844 is solvent exposed and may specifically stabilize the active form by interacting with two acidic residues, one of which resides in a different domain. We analyzed the evolutionary prevalence of the Gln844Arg mutation, finding that Cas9 homologs predominantly exhibit the wild type Gln identity (80% compared to only 16% for Arg; Supplementary Fig. 10b). Thus, even mutations that occur in the minority of homologs may have a beneficial impact on activity.

The other four mutations in TurboCas9 (Val842Leu, Phe846Tyr, Leu847Met, and Ile852Phe) optimize core packing and hydrogen bonding interactions within the Cas9 enzyme (Supplementary Fig. 10a). Such mutations may indirectly stabilize the catalytically competent form of the enzyme to increase its efficiency. Thus, the combination of phylogenetic and atomistic design calculations that forms the basis for FuncLib calculations focuses design calculations on a small number of constructs. Our modeling suggests that the designed mutations stabilize the HNH domain and its interactions with the Arg domain in the catalytically active conformation of Cas9.

## Discussion

In summary, the computational design of the Cas9 HNH domain enabled the production of hyperactive Cas9 enzymes with the ability to introduce larger and more extensive mutational signatures. A few amino acid positions that were targeted by FuncLib were previously targeted for alanine scanning mutagenesis by Slaymaker et al.^19^ to reduce off-target effects, rather than to increase the on-target activity. Interestingly, Slaymaker et al.^19^ also designed a number of mutations to decrease specificity by strengthening the interaction of Cas9 with the non-target strand, with the main one of interest being an L847R substitution. Our design 2.1 and TurboCas9 both have this position substituted with a methionine instead of an arginine and in the case of TurboCas9, we observed no overall changes in off-target activity.

The combinatorial layering of activity-enhancing variants resulted in epistatic effects in our study, and in some cases increasing activity when two designed regions were combined. Interestingly, two of the four best single region designs are from region 2 which is centered on H840 of the HNH active site. The other two are from regions 1 and 3 which make up the hinges of the HNH domain, supporting its dynamic repositioning^20,21^. Furthermore, all our combined designs that have been verified in both mammalian and yeast settings comprise a region 2 and either a region 1 or region 3 mutant. This suggests that for enhanced Cas9 on-target activity and perhaps tolerable off-target activity, having mutations in region 2 is essential and can be potentially further enhanced by adding mutations in either region 1 or 3. This result and our discovery of numerous hyperactive Cas9 enzymes verify the hypothesis that the HNH domain is not naturally optimized for DNA cleavage efficiency. This might reflect the competing evolutionary pressures on natural Cas9 enzymes to balance activity, specificity and Cas9 abundance relative to tracrRNA, crRNAs, and their target DNAs, in their roles of targeting viral genomes for destruction as well as PAM-specific spacer acquisition^22^.

Structural studies have shown that Cas9 positions its gRNA and target DNA prior to reorientation of the HNH domain for cleavage^20^. Displacement of the non-target strand and R-loop formation then enable cleavage by the HNH domain^5,21^. For designs with the same number of cleaved alleles but more extensively mutated targets (such as design 1.4) it could be that the introduced mutations enhance cleavage without improving R-loop formation, while for others (such as TurboCas9) both binding and cleavage might be enhanced, or alternatively TurboCas9 might bind less strongly after cleavage, thereby exposing the DNA to more diverse repair outcomes.

Since the discovery of CRISPR gene editing much attention has focused on improving the specificity of Cas proteins^23^; however, for many applications efficiency could be more important than specificity. We recommend using TurboCas9 where multiple genes need to be targeted simultaneously, for example in cancer, in which multiple oncogenes might need to be disabled to halt cancer cell growth. The higher activity would also enable applications where multiple cleavage events would be required, for example in vitro applications using Cas9 in a way more analogous to a restriction enzyme^24^, or in other situations where cleavage efficiency might be limiting. The FuncLib designed hyperactive Cas9 enzymes described here provide tools to address these gaps. Furthermore, the ability of these enzymes to introduce more extensive deletions and complex repair scars from multiple edits could be useful to more effectively knockout genes or to provide diverse signatures for cellular recording and lineage tracing^25^.

## Methods

### Computational enzyme design

We performed three separate FuncLib^13^ runs, independently mutating regions including amino acids 765–780, 838–853, and 911–924 of SpCas9 using PDB:5F9R as a structural model^21^. The sequence space for each position in regions 1–3 was explored using the combination of PSSM and Rosetta energy filters. The protein elements from ~100,000 designs were explicitly modeled for each region, each with 3–5 mutations from the parental Cas9. The catalytic histidine (H840) was fixed during the production of the region 2 designs. All designs were ranked according to the all-atom Rosetta energy function^26^, which is dominated by van der Waals packing, solvation, electrostatics and hydrogen bonding, interactions with DNA and metal ions were not included in energy calculations. The best ten designs of each region, which differed by at least 3 mutations from one another, were selected for experimental testing.

### Plasmid construction

SpCas9 was codon optimized for *S. cerevisiae* using Gene Designer software (ATUM) and synthesized by Integrated DNA Technologies (IDT). Wild-type Cas9 was designed in four gBlocks and assembled using Gibson assembly in pJ201 (ATUM). The Cas9 ORF was sub-cloned into the yeast expression plasmids pCM251 and pCM252 using BamHI and NotI. The three regions of the HNH domain that were selected for Funclib mutagenesis were flanked by SpeI-BsaI, BsmBI-SacII and XbaI-StuI restriction sites, respectively. Each region containing the mutations of interest was designed in Gene Designer and synthesized by Twist Biosciences. Designed regions were either individually cloned into Cas9 or as combinations. The pRS426-*CAN1* gRNA plasmid^27^ (Addgene #43803) was used for gRNA expression in yeast and two separate gRNAs targeting *ADE2* and *HIS3* were synthesized by IDT and sub-cloned in place of the *CAN1* gRNA using NheI and MluI. The Cas9 inhibitors AcrIIA2 and AcrIIA4 fused with a P2A peptide and flanked by the *CUP1* promoter and *PGI1* terminator were synthesized by IDT and cloned into pRS426 gRNA plasmids using KpnI and MluI. For experiments in human cells, the designed HNH domains were human codon optimized and subcloned into the mammalian Cas9 expression plasmid pD1311-AD (ATUM) using the restriction enzymes SacI and SphI. Cassettes for expressing the various gRNAs were produced as gBlocks (IDT) and subcloned into pD1311-AD using BbsI. All plasmids were verified using Sanger sequencing by the Australian Genomics Research Facility (AGRF), Perth, Western Australia. Sequences of designed open reading frames are provided in Supplementary Table 1.

### Yeast transformation and survival assays

*Saccharomyces cerevisiae* BY4738 cells (MATα *trp1Δ63 ura3Δ0*)^28^ were obtained from Jef Boeke, New York University. Cells were transformed with plasmids using the LiAc method according to Gietz and Woods^29^, plated on SC-T-U, and incubated at 30 **°**C for 2–3 days. A single colony was grown overnight in 10 ml of SC-T-U media at 30 °C. Yeast cultures were standardized to one OD600 in TE and three serial 1/10 dilutions were made in TE. Of each dilution, 5 μl were plated out on selective media (SC) with the appropriate nutrients lacking and supplemented with anhydrotetracycline and canavanine where indicated. Plates were grown for 2–3 days at 30 °C until adequate growth was observed for the control yeast samples on each plate. For quantitative survival assays, single colonies were grown overnight in 10 ml of SC-T-U media at 30 °C. Cells were standardized to one OD_600_ and diluted to 2.8 × 10^-3^ in TE. Of each sample, 100 μl were plated on selective media with or without anhydrotetracycline lacking the appropriate auxotrophic nutrients and grown for 2 days at 30 °C.

### Mammalian cell transfection and amplicon sequencing

HEK293T (CRL-3216) and HeLa (CCL-2) cells were obtained from ATCC and cultured at 37 °C under humidified 95% air/5% CO_2_ in Dulbecco’s modified Eagle’s medium (DMEM) containing glucose (4.5 g/l), 1 mM sodium pyruvate, 2 mM glutamine and 10% fetal bovine serum (FBS). Cells were seeded at 60% confluence in 24-well plates and transfected after 24 h with 500 ng of DNA in OptiMEM media (Gibco, Life Technologies) using a 1:1 mix of two transfection reagents (Lipofectamine LTX Reagent with Plus Reagent, Invitrogen, and FuGene HD, Promega)^30^. Each Cas9 variant was transfected in triplicate. For HDR experiments, a short single-stranded homologous DNA was co-transfected in a total amount of 500 ng of DNA including Cas9 plasmid. 72 h after transfection the cells were trypsinized and the cell pellets lysed for DNA extraction using the KAPA Express Extract Kit, according to the manufacturer’s instructions (Sigma-Aldrich). Amplicons were generated using primers flanking the gRNA and incorporating Illumina adaptor sequences (Supplementary Table 2). For long amplicon deep sequencing, PCR produced were converted into sequencing libraries by tagmentation (Illumina). Libraries were sequenced on an Illumina MiSeq using 250 bp paired-end chemistry by the Australian Genomics Research Facility (AGRF), Perth, Western Australia.

### Next generation sequencing analyses

Sequenced reads were trimmed with TrimGalore^31^ (v0.6.6) using cutadapt^32^ (v1.18) and fastqc^33^ (v0.11.9) (--paired –nextera –fastqc). Trimmed reads were merged with FLASH^34^ (v1.2.11) (--min-overlap 10 –max-overlap 250) and aligned to the amplicon sequences with BLAT^35^ (v37x1) (-minScore = 0 -stepSize = 1 -out = psl). To avoid a heterozygous genomic insertion that complicated interpretation of the CIGAR string, 23 nt were trimmed from 3′ end of *MRPL45* reads with TrimGalore (--three_prime_clip_R1 23). To avoid a 5′ terminal nucleotide next to the adapter region that complicated interpretation of the CIGAR string, 1 nt was trimmed from 5′ end of *BCL11A* chr10 off target reads with TrimGalore (--clip_R1 1) after merging. The resultant .psl file was converted to SAM/BAM format with the uncle_psl.py script (https://github.com/bsipos/uncle_psl). The resulting BAM files were parsed with command-line tools based on the number of alphabetic characters in the CIGAR sequence (which we have termed CIGAR complexity). Since these characters represent specific alignment characteristics (match, insertion, deletion, or soft-clipping) and are paired with a number describing their length, we used this information to determine the lengths and locations of deletion and insertion events for all alignments. Alignments that contained soft-clipped sequences, or with a CIGAR complexity of 7 or above, were excluded. All configurations of alignment up to a CIGAR complexity of 6 and the simplest of complexity 7 (MIDMIDM) were collated and summarized. Sequenced reads were trimmed with TrimGalore^27^ (v0.6.6) using cutadapt^29^ (v1.18) and fastqc^30^ (v0.11.9) (--paired --nextera --fastqc). Trimmed reads were aligned to amplicon sequences with Bowtie2^36^ v2.4.4 with default parameters, and variants called with freebayes^37^ v1,3,4 with ploidy (-p) set to 3 for *BCL11A* or 5 for *VEGFA*, and the parameters: -F 0.01 --haplotype-length 20 --pooled-continuous.

For long amplicon deep sequencing analyses adaptor trimming was performed using Trimmomatic 0.39^38^ and aligned with Hisat2 in paired-end mode with no-spliced-alignment being permitted and with read gap open/close set to 1,1 and reference gap open/close set to 10000, 1000^39^. The resultant alignments were parsed using Python3 script CIGAR strings, which were used to identify any read pairs having soft slipping or deletions. Deletions were taken as called, however, where soft clipping was performed the soft clipped end was searched for in prior bases with string length starting from 30 and reducing to 5. Where a unique match was observed this was taken as evidence of a large deletion. The resultant read pairs were then aligned via a custom Python script (https://github.com/ORAFLAB/DeletionQuantifier) and those reads whose mapping or deletion coordinates included any positions within the gRNA (positions 2059–2078 within the reference amplicon sequence) were retained for display.

### Mathematical analysis of gene editing results

$${{{{{\rm{CFU}}}}}}\,({{{{{\rm{Standardized}}}}}}\; {{{{{\rm{to}}}}}}\;100)=\frac{{raw}\,{sample}\,{CFU}}{{raw}\,{CFU}\,{from}\,{control}\,{plate}/100}$$

Variables:

Raw sample CFU = raw colony counts from plates missing either adenine or histidine

Raw CFU from control plate = raw colony counts from loading control plates

CFU (Standardized to 100) = normalized CFU

$${{{{{\rm{CFU}}}}}}\,({{{{{\rm{relative}}}}}}\; {{{{{\rm{to}}}}}}\; {{{{{\mathrm{WT}}}}}})=\frac{{normalised}\; {CFU}}{{average}\; {{{{{\rm{CFU}}}}}}\; {{{{{\rm{of}}}}}}\; {{{{{\rm{WT}}}}}}}$$

Variables:

Normalized CFU = normalized CFU standardized to a CFU of 100

Average CFU of WT = average of the normalized counts for WT Cas9

CFU (relative to WT) = Relative CFU compared to WT

$${{{{{\rm{Fold}}}}}}\; {{{{{\rm{Change}}}}}}\,({{{{{\rm{relative}}}}}}\; {{{{{\rm{to}}}}}}\; {{{{{\rm{WT}}}}}})=\left(\frac{{normalized}\,{sampl}e\,{CFU}}{{average}\,{control}\,{CFU}}-1\right)* {average}\left(\frac{{CFU}\,{WT}\;{{{{{\rm{\#}}}}}}n}{{average}\,{WT}\,{CFU}}\right)$$

Variables:

Normalized sample CFU = normalized CFU standardized to a CFU of 100

Average control CFU = average of the normalized counts for control

CFU WT #*n* = relative CFU for WT sample 1 through 3

Average WT CFU = average of the normalized counts for WT Cas9

$${{{{{\rm{Average}}}}}}\; {{{{{\rm{Length}}}}}}\; {{{{{\rm{of}}}}}}\; {{{{{\rm{Indels}}}}}}=\frac{{sum}({Read}\; {count}\; {for}\; {n}\; {bp}\; {indels}* {n}\; {bp})}{{total}\; {number}\; {of}\; {reads}\; {for}\; {indel}\; {t}{ype}}$$

Variables:

Average length of Indels = average length of different indels in bp

Total number of reads for indel type = sum of all reads for indel type

$${{{{{\rm{Fidelity}}}}}}\; {{{{{\rm{TurboCas}}}}}}9=\; {{{{{\rm{Average}}}}}}\left(1-\frac{{Mutant}\; {read}\; {count}\; {for}\; {TurboCas}9\,n{{{{{\rm{\#}}}}}}}{100000}\right)\Big/ \\ \left({average}\left(1-\frac{{Edited}\,{read}\,{count}\,{for}\,{WT}\,{Cas}9\,n{{{{{\rm{\#}}}}}}}{100000}\right)\right)$$

$${{{{{\rm{Fidelity}}}}}}\; {{{{{\rm{WTCas}}}}}}9=\, {{{{{\rm{Average}}}}}}\left(1-\frac{{Edited}\,{read}\,{count}\,{for}\,{WT}\,n{{{{{\rm{\#}}}}}}}{100000}\right)\Big/ \\ \left({average}\left(1-\frac{{Edited}\,{read}\,{count}\,{for}\,{WT}\,{Cas}9\,n{{{{{\rm{\#}}}}}}}{100000}\right)\right)$$

Variables:

Edited read count for TurboCas9 n# = number of edited reads for TurboCas9 sample 1 through 3

Edited read count for WT Cas9 n# = number of edited reads for WT Cas9 sample 1 through 3

Fidelity = Fidelity of TurboCas9 relative to WT Cas9 and WT Cas9

### Statistical analysis

Statistical significance of yeast survival assay results were calculated using a standard Student’s *t*-test with two-tailed distribution and unequal variance was assumed between samples. An FDR-adjusted Student’s *t*-test, using the same parameters, was used to calculate statistical significance in the mammalian gene editing experiments.

### Reporting summary

Further information on research design is available in the Nature Research Reporting Summary linked to this article.

## Supplementary information

Supporting Information

Reporting Summary

## Data Availability

The authors declare that the data supporting the findings of this study are available within the paper and its supplementary information files. Deep sequencing data are available at the NCBI Sequence Read Archive (SRA) under BioProject ID PRJNA762160. Source data are provided in this paper.

## Source data

Source data
