# Supplementary material for: Computationally designed hyperactive Cas9 enzymes
Source: Nat Commun. 2022 May 31;13:3023. doi: 10.1038/s41467-022-30598-9 (PMC9156780; doi:10.1038/s41467-022-30598-9)
Supplement: Supplementary file 1 — Supporting Information [file 41467_2022_30598_MOESM1_ESM.pdf]

Supplementary Information:
Computationally designed hyperactive Cas9 enzymes

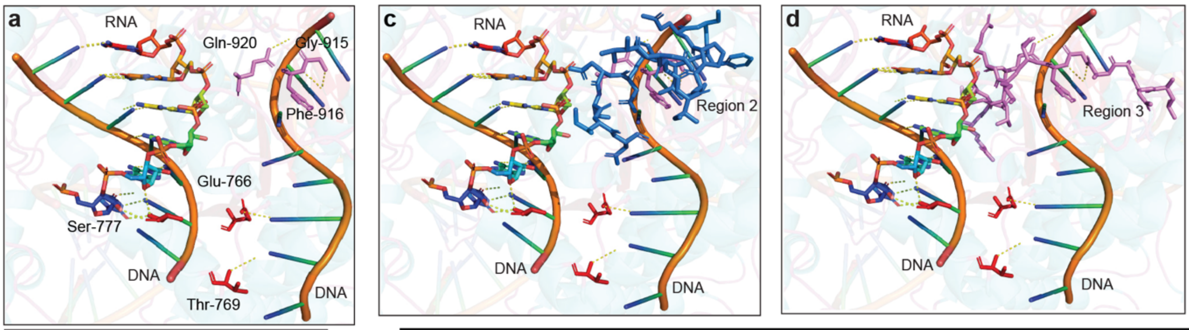

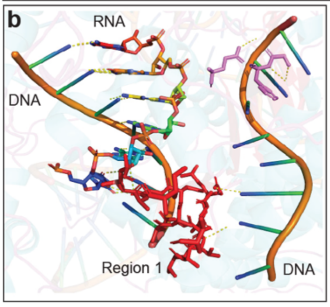

e

	Amino acid substitution		
	Region 1	Region 2	Region 3
Design 1	Q771E, G773K, Q774R, K775Q, R780K	Q844R, L847M, K848T, D850N, I852F	K913R, F916W, R919Q, V922I, E923D
Design 2	Q771R, K772Q, G773K, K775H, R780K	V842L, Q844R, F846Y, L847M, I852F	G915T, F916W, I917L, K918E, R925Q
Design 3	N767E, Q771K, K775N, N776Q, R780K	V838I, Q844R, L847T, K848T, I852L	A914Q, G915H, F916W, V922I, R925Q
Design 4	N767E, Q771R, K772Q, K775D, R780K	V842I, Q844R, K848R, D849N, I852L	A914Q, G915H, F916Y, I917L, V922I
Design 5	E766D, N767E, Q771G, K772E, R780K	Q844R, L847M, I852V, D853N	L911S, K913Q, A914Q, G915N, F916W
Design 6	E766D, N767E, G773K, N776Q, R780K	V842I, Q844R, K848V, D850N, I852F	A914Q, G915H, F916W, I917V, L921I
Design 7	R765K, N767E, K772Q, G773K, Q774R	V842I, S845A, L847M, K848V, I852F	K913E, A914Q, G915R, F916W, V922I
Design 8	Q771E, K775R, R780K	Q844R, D850N, S851T, I852F	D912E, A914Q, I917V, V922M
Design 9	R765K, Q771N, K772Q, G773K, R780K	Q844R, F846Y, L847M, K848R, I852V	K913E, A914Q, G915R, F916W, R925Q
Design 10	R765K, E766Q, K772E, G773K, R780K	I841V, V842I, L847M, K848T, D853E	K913E, G915R, F916W, I917V, V922M

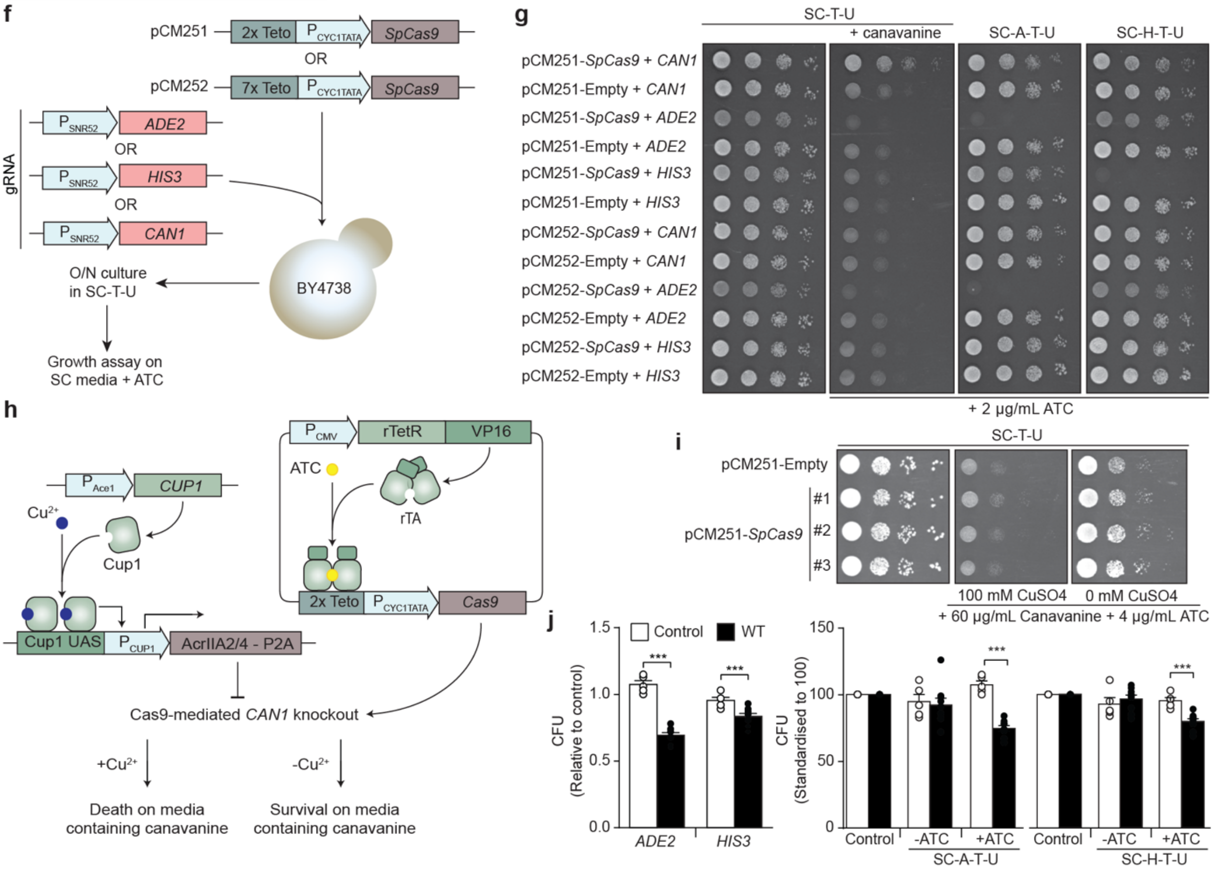

Supplementary Fig. 1 A yeast system for the quantification of FuncLib designs. (**a-d**) Close-up views of the Cas9 HNH domain (**a**)(PDB accession code 5F9R, https://www.wwpdb.org/pdb?id=pdb_00005f9r)[21], highlighting region 1 (**b**), region 2 (**c**), and region 3 (**d**) that were targeted for computational mutagenesis. (**e**) Sequences of the 10 best designs for each region, as determined by total protein energy scoring. (**f**) Schematic

representation of the vectors and gRNAs used in our approach that harnesses yeast to screen for active Cas9 mutants. (**g**) Yeast survival assays examining the functionality of Cas9 expression plasmids and the control (empty vector) with gRNAs targeting *ADE2* (media lacking adenine, SC-A-T-U), *HIS3* (media lacking histidine, SC-H-T-U) and *CAN1* genes (media containing canavanine, SC-T-U + canavanine). Initially, we used two different yeast expression plasmids, pCM251 and pCM252, which differ only in the number of tetracycline-responsive operators, 2 and 7 respectively. Induction of Cas9 by anhydrotetracycline (ATC) was found to be less robust when using pCM252, therefore all experiments done hereafter were performed with pCM251. (**h**) Schematic representation of quantitative Cas9 assay system. To prevent premature induction of Cas9 activity we introduced two well-characterized Cas9 inhibitors, AcrIIA2 and AcrIIA4, which bind in distinct modes within the Cas9-gRNA complex to block its activity. AcrIIA2 and AcrIIA4 are fused by a self-cleaving peptide (P2A) and their expression controlled with a copper-inducible promoter (*CUP1*) and cloned into the gRNA plasmid. The addition or subtraction of ATC and copper sulfate was used to tightly control Cas9 activity. (**i**) Yeast survival assay using the Cas9 inhibitor system with a gRNA targeting *CAN1* and canavanine as the selective agent. (**j**) Quantification of Cas9-mediated gene knockout upon repression or induction of AcrIIA2 and AcrIIA4 inhibitors, compared to a negative control lacking Cas9 (empty vector). Survival in the absence of adenine with a gRNA targeting *ADE2* or survival in the absence of histidine with a gRNA targeting *HIS3*, are shown in the left and right portions of each graph, respectively. CFU (colony forming units), error bars: s.e.m. of n=9 biologically independent replicates. A standard Student's *t*-test with a two-tailed distribution and unequal variance assumed between samples was used to calculate the significance. p-values: *** p<0.001. Specific p-values for WT vs Empty for *ADE2* p=1.8e-05 and *HIS3* p=9.0e-04 (Student's t-test). Source data are provided as a Source Data file.

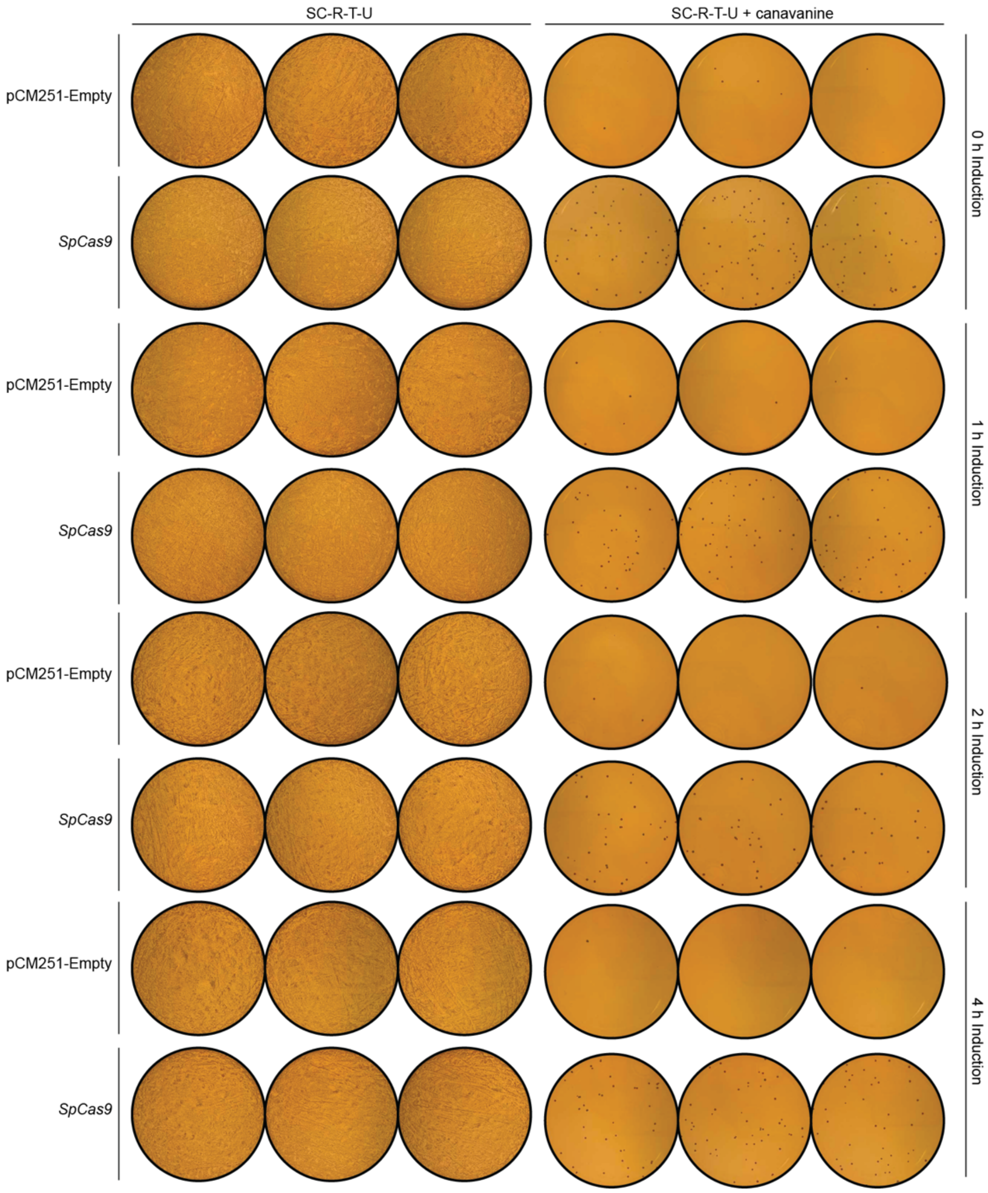

Supplementary Fig. 2 Gene editing is inducible in the absence of copper-induced expression of Cas9 inhibitors. Overnight cultures of yeast grown in SC media lacking arginine and grown for either 0, 1, 2 or 4 h in media lacking arginine, then plated on media supplemented with canavanine or without, to establish cell viability. All plates were supplemented with ATC and all lacked arginine. Source data are provided as a Source Data file.

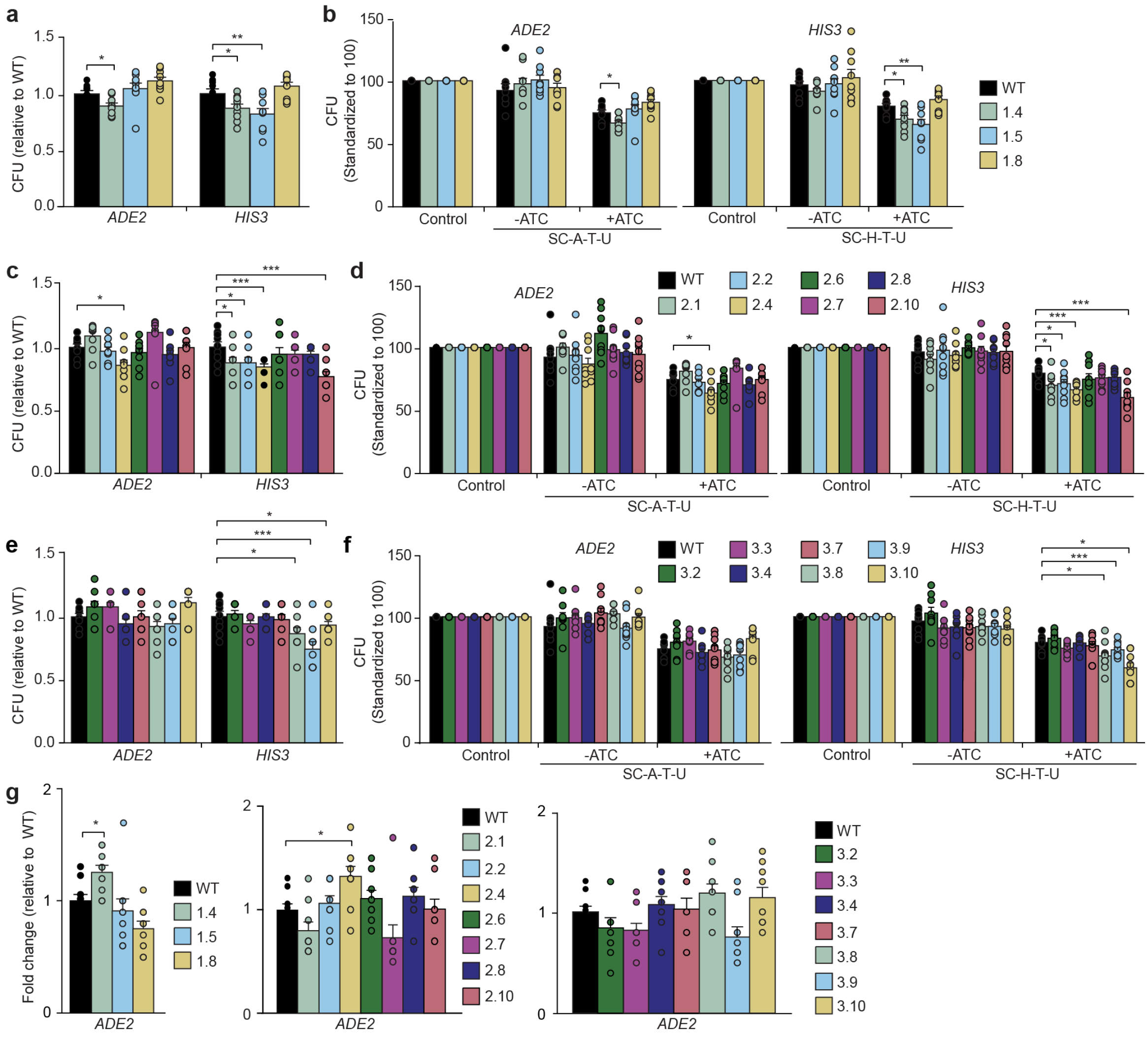

Supplementary Fig. 3 Quantification of individual FuncLib designs in yeast. Quantification of engineered Cas9 activity via yeast survival with the Cas9 inhibitor system and gRNAs targeting *ADE2* and *HIS3*. (**a**) Activity of individual designs for region 1 in colony forming units (CFU) relative to wild-type (WT) Cas9; and (**b**) standardized to 100 for comparison. (**c**) Activity of individual designs for region 2 in CFU relative to WT Cas9; and (**d**) standardized to 100 for comparison. (**e**) Activity of individual designs for region 3 in CFU relative to WT Cas9; and **f** standardized to 100 for comparison. (**g**) Fold change relative to WT for the *ADE2* gRNA, quantification for active designs for all regions. Error bars: s.e.m. of n=9 biologically independent replicates. A standard Student's *t*-test with a two-tailed distribution and unequal variance assumed between samples was used to calculate the significance. p-values: * p<0.05, ** p<0.01, *** p<0.001. Specific p-values for panel (**b**) for design vs WT targeting *HIS3*: 1.4 p=0.01, 1.5 p=0.008; for design vs WT targeting *ADE2*: 1.4 p=0.01 (Student's t-test). Specific p-values for panel (**d**) for design vs WT targeting *HIS3*: 2.1 p=0.02, 2.2 p=0.05, 2.4 p=0.0003, 2.10 p=0.0007; for design 2.5 vs WT targeting *ADE2* p=0.01 (Student's t-test). Specific p-values panels (**e**,**f**) for design vs WT targeting *HIS3*: 3.8 p=0.02, 3.9 p=0.05 and 3.10 p=0.0006 (Student's t-test). Specific p-values for panel (**g**) for design vs WT targeting *ADE2*: 1.4 p=0.01 and 2.4 p=0.01 (Student's t-test).

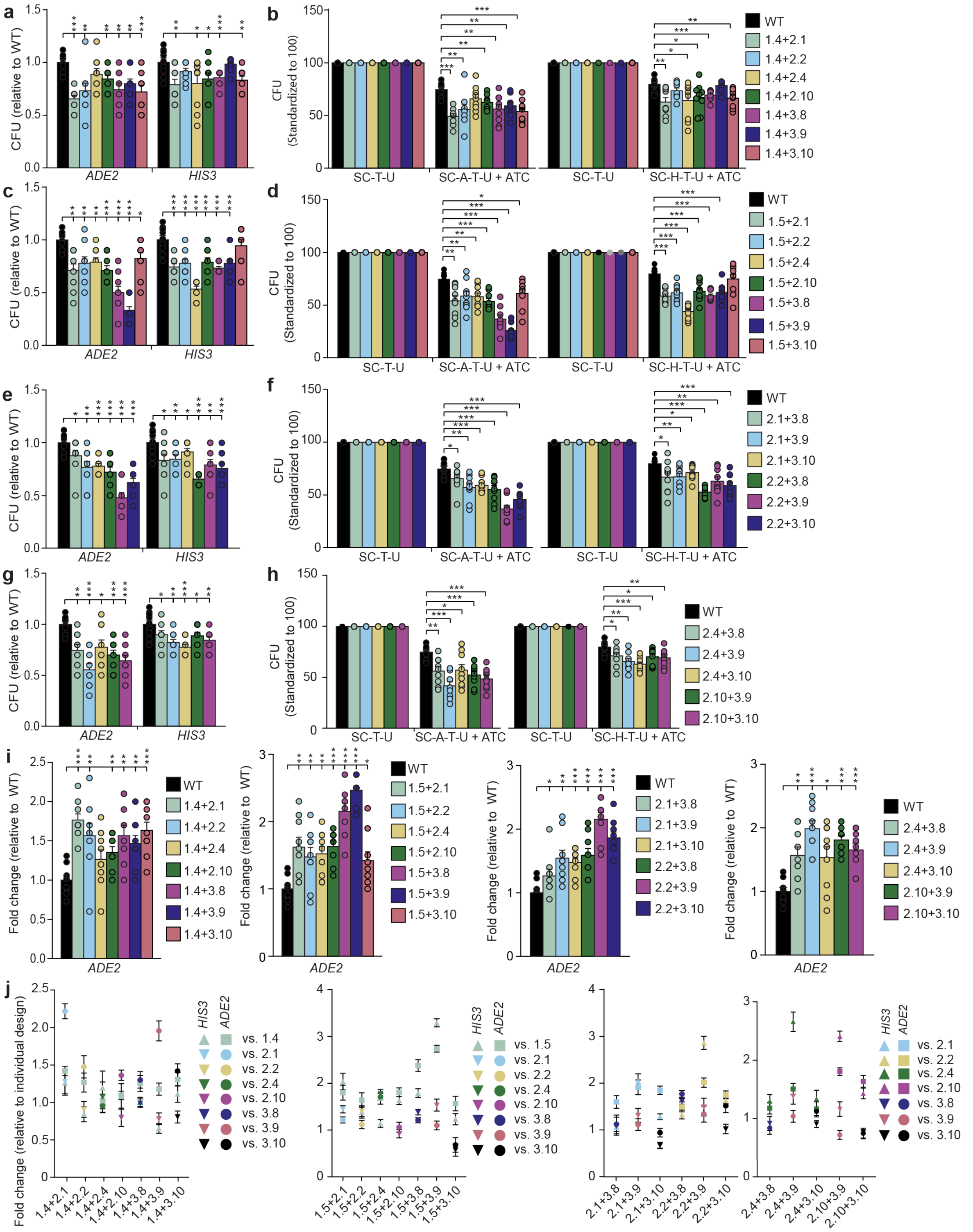

Supplementary Fig. 4 Quantification of FuncLib double mutants in yeast. (**a**) Activity of combined designs containing design 1.4 targeting *ADE2* and *HIS3* in colony forming units (CFU) relative to wild-type (WT) Cas9; and (**b**) standardized to 100 for comparison. (**c**) Activity of combined designs paired with mut 1.5 in CFU relative to WT Cas9; and (**d**) standardized to 100 for comparison. (**e**) Activity of combined designs that include mut 2.1 and mut 2.2 sequences in CFU relative to WT Cas9; and (**f**) standardized to 100 for comparison. (**g**) Activity of combined designs that contain design 2.4 and 2.10 sequences in CFU relative to WT Cas9; and (**h**) standardized to 100 for comparison. (**i**) Fold change relative to WT for *ADE2* gRNA, quantification for active combined designs. (**j**) Comparison of

combined designs' activity relative to their individual counterparts. Fold change relative to individual designs for *ADE2* and *HIS3* gRNAs. Error bars: s.e.m. for n=9 biologically independent replicates. A standard Student's *t*-test with a two-tailed distribution and unequal variance assumed between samples was used to calculate the significance. p-values: * p<0.05, ** p<0.01, *** p<0.001. Specific p-values for panels (**a**,**b**) *HIS3*: 1.4+2.1 p=0.004, 1.4+2.4 p=0.03, 1.4+2.10 p=0.03, 1.4+3.8 p=0.001, 1.4+3.10 p=0.002; *ADE2*: 1.4+2.1 p=1.4E-06, 1.4+2.2 p=0.006, 1.4+2.10 p=0.001, 1.4+3.8 p=0.003, 1.4+3.9 p=0.001, 1.4+3.10 p=0.0003 (Student's t-test). Specific p-values for panels (**c**,**d**) *HIS3*: 1.5+2.1 p=1.4e-06, 1.5+2.2 p=8.2e-05, 1.5+2.4 p=4.9e-09, 1.5+2.10 p=6.2e-04, 1.5+3.8 p=6.7e-07, 1.5+3.9 p=1.4e-04; *ADE2*: 1.5+2.1 p=2.1e-03, 1.5+2.2 p=9.7e-03, 1.5+2.4 p=1.4e-03, 1.5+2.10 p=2.2e-05, 1.5+3.8 p=1.5e-06, 1.5+3.9 p=1.9e-11, 1.5+3.10 p=1.9e-02 (Student's t-test). Specific p-values for panels (**e**,**f**) *HIS3*: 2.1+3.8 p=0.04, 2.1+3.9 p=0.005, 2.1+3.10 p=0.02, 2.2+3.8 p=2.5e-08, 2.2+3.9 p=0.004, 2.2+3.10 p=0.0002; *ADE2*: 2.1+3.8 p=0.05, 2.1+3.9 p=0.003, 2.1+3.10 p=0.0001, 2.2+3.8 p=0.0006, 2.2+3.9 p=3.7e-07, 2.2+3.10 p=7.0e-07 (Student's t-test). Specific p-values for panels (**g**,**h**) *HIS3*: 2.4+3.8 p=0.04, 2.4+3.9 p=0.001, 2.4+3.10 p=3.2e-05, 2.10+3.9 p=0.01, 2.10+3.10 p=0.009; *ADE2*: 2.4+3.8 p=0.003, 2.4+3.9 p=1.5E-05, 2.4+3.10 p=0.01, 2.10+3.9 p=8.2e-05, 2.10+3.10 p=1.5e-05. Specific p-values for panels (**i**,**j**): 1.4+2.1 p=1.4e-06, 1.4+2.2 p=0.006, 1.4+2.10 p=0.001, 1.4+3.8 p=0.003, 1.4+3.9 p=0.001, for 1.4+3.10 p=0.0003, 1.5+2.1 p=2.1e-03, 1.5+2.2 p=9.7e-03, 1.5+2.4 p=1.4e-03, 1.5+2.10 p=2.2e-05, 1.5+3.8 p=1.5e-06, 1.5+3.9 p=1.9e-11, 1.5+3.10 p=1.9e-02, 2.1+3.8 p=0.05, 2.1+3.9 p=0.003, 2.1+3.10 p=0.0001, 2.2+3.8 p=0.0006, 2.2+3.9 p=3.7e-07, 2.2+3.10 p=7.0e-07, 2.4+3.8 p=0.003, 2.4+3.9 p=1.5e-05, 2.4+3.10 p=0.01, 2.10+3.9 p=8.2e-05, 2.10+3.10 p=1.5e-05 (Student's t-test).

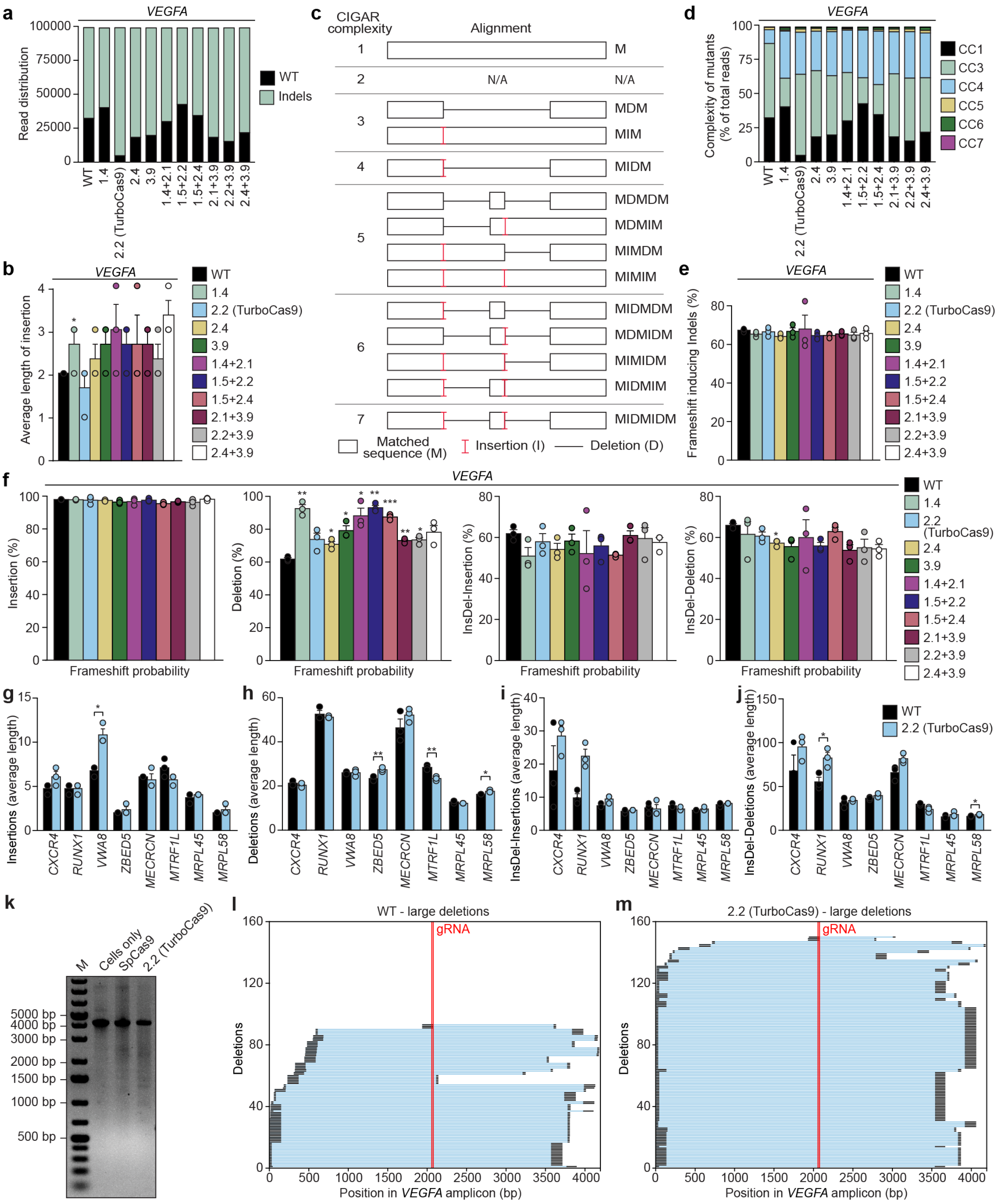

Supplementary Fig. 5 Complexity of mutations introduced by engineered Cas9 enzymes in human cells. (a) The distribution of WT and indel-containing alleles within samples. (**b)** Average insertion lengths in each sample. (**c)** CIGAR complexity levels of mutations introduced into the *VEGFA* gene are shown schematically. CIGAR complexity (CC) level 1 comprises all full length aligned wild-type sequences, CC2 are all soft clipped reads which were excluded from our analysis. CC3 are single insertion or deletion event and CC4 contains combined events with a single deletion and insertion. CC5 and above are of increasing complexity and comprise alleles with deletions and insertions occuring simultaneously, in varying numbers and in different combinations. (**d)** Distribution of the

different CC levels in *VEGFA* alleles upon editing by engineered Cas9 enzymes. (**e**) Occurrence of indels that cause a frameshift. (**f)** Occurrence of mutations that cause a frameshift, classified by a particular mutation type. **(g-j)** Average length of **(g)** insertions, (**h**) deletions, (**i**) InsDel-insertions and (**j**) InsDel-deletions. (**k**) Agarose gel electrophoresis of long amplicons for *VEGFA*. M denotes the molecular weight marker. (**l,m**) Graphical representation of deletions occurring within the long *VEGFA* amplicon for cells expressing wild-type Cas9 (**l**) and design 2.2 (TurboCas9) (**m**), detected by deep sequencing. Deletions >800 bp are shown schematically. Error bars: s.e.m. of n=3 biologically independent replicates. An FDR-adjusted Student's *t*-test with a two-tailed distribution and unequal variance assumed between samples was used to calculate the significance. P-value: * p<0.05, ** p<0.01, *** p<0.001. Specific p-value for panel (**b**) for design 1.4 vs wild-type Cas9 p=0.05 (FDR-adjusted Student's t-test). Specific p-values for panel (**f**) for frameshift probability from deletions for design vs wild-type Cas9: 1.4 p=0.01, 2.4 p=0.03, 3.9 p=0.03, 1.4+2.1 p=0.03, 1.5+2.2 p=0.001, 1.5+2.4 p=0.001, 2.1+3.9 p=0.002, 2.2+3.9 p=0.01; for frameshift probability from InsDel-deletions for design 2.4 vs wild-type p=0.02 (FDR-adjusted Student's t-test). Specific p-value for panel (**g**) for TurboCas9 vs wild-type for *VWA8* p= 0.02 (FDR-adjusted Student's t-test). Specific p-values for panel (**h**) for TurboCas9 vs wild-type: *ZBED5* p= 0.008, *MTRF1L* p= 0.004, *MRPL58* p= 0.01 (FDR-adjusted Student's t-test). Specific p-value for panel (**i**) for TurboCas9 vs wild-type Cas9 for *RUNX1* p= 0.02 (FDR-adjusted Student's t-test). Source data are provided as a Source Data file.

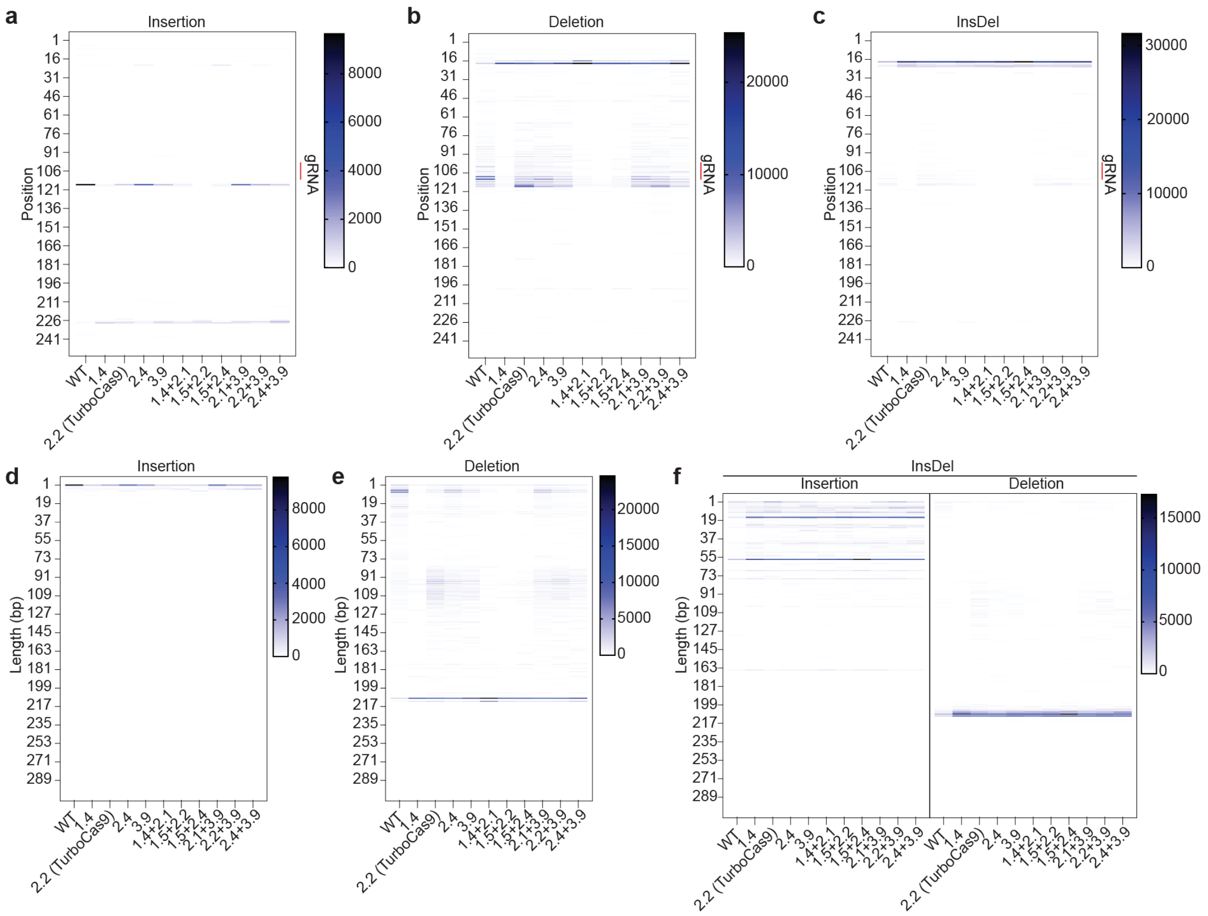

Supplementary Fig. 6 Positioning and length characteristics of mutations introduced in human cells. (**a-c**) Heatmaps summarizing the positions of mutations in sequencing data. Positional data for the three general classes of editing events at the *VEGFA* locus: insertions (**a**), deletions (**b**), and combined insertion-deletion events (**c**). Scale bars depict read counts. Location of the gRNA binding site is shown as a red bar. (**d-f**) Length distribution of indels.

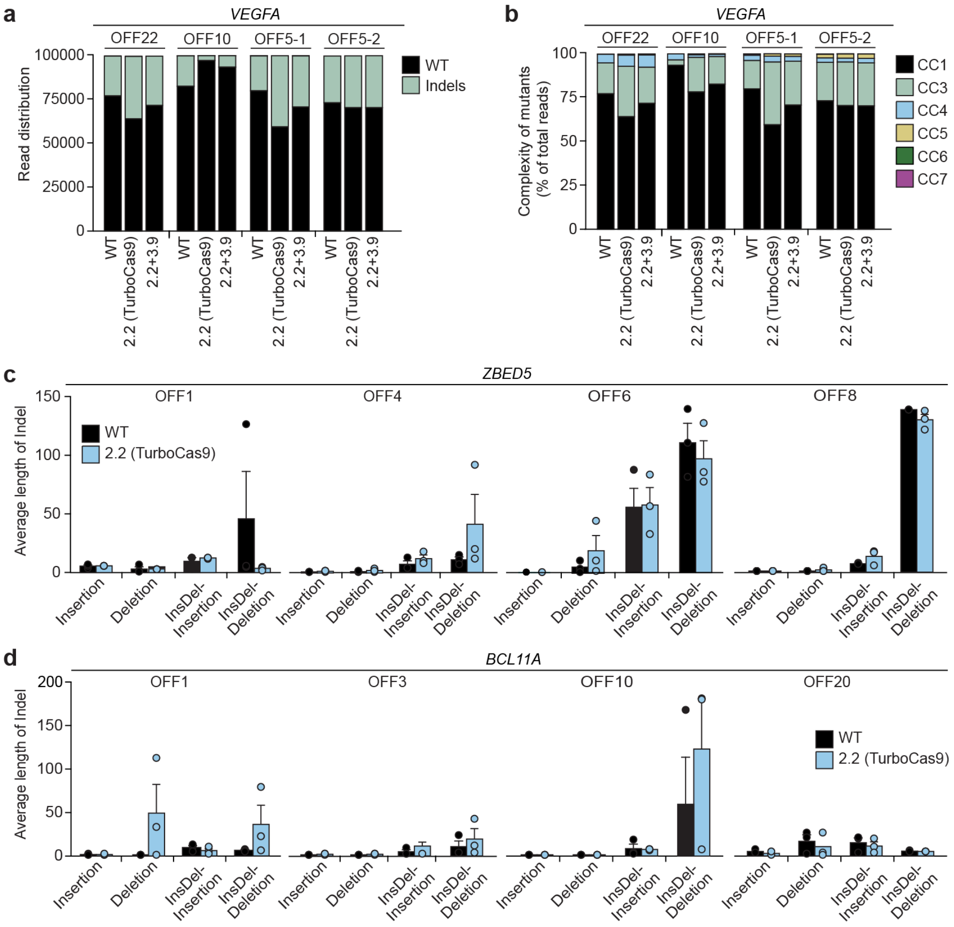

Supplementary Fig. 7 Enhanced Cas9 on-target activity does not necessarily increase off-target activity. (**a**) Read distribution of WT compared to mutated alleles following gene editing by selected mutants relative to wild-type Cas9. (**b)** CIGAR complexity (CC) level of edits introduced into off-target sites. (**c-d**) Average length of four indel types for the off-targets of (**c**) *ZBED5* and (**d**) *BCL11A*. Error bars: s.e.m. for n=3 biologically independent replicates.

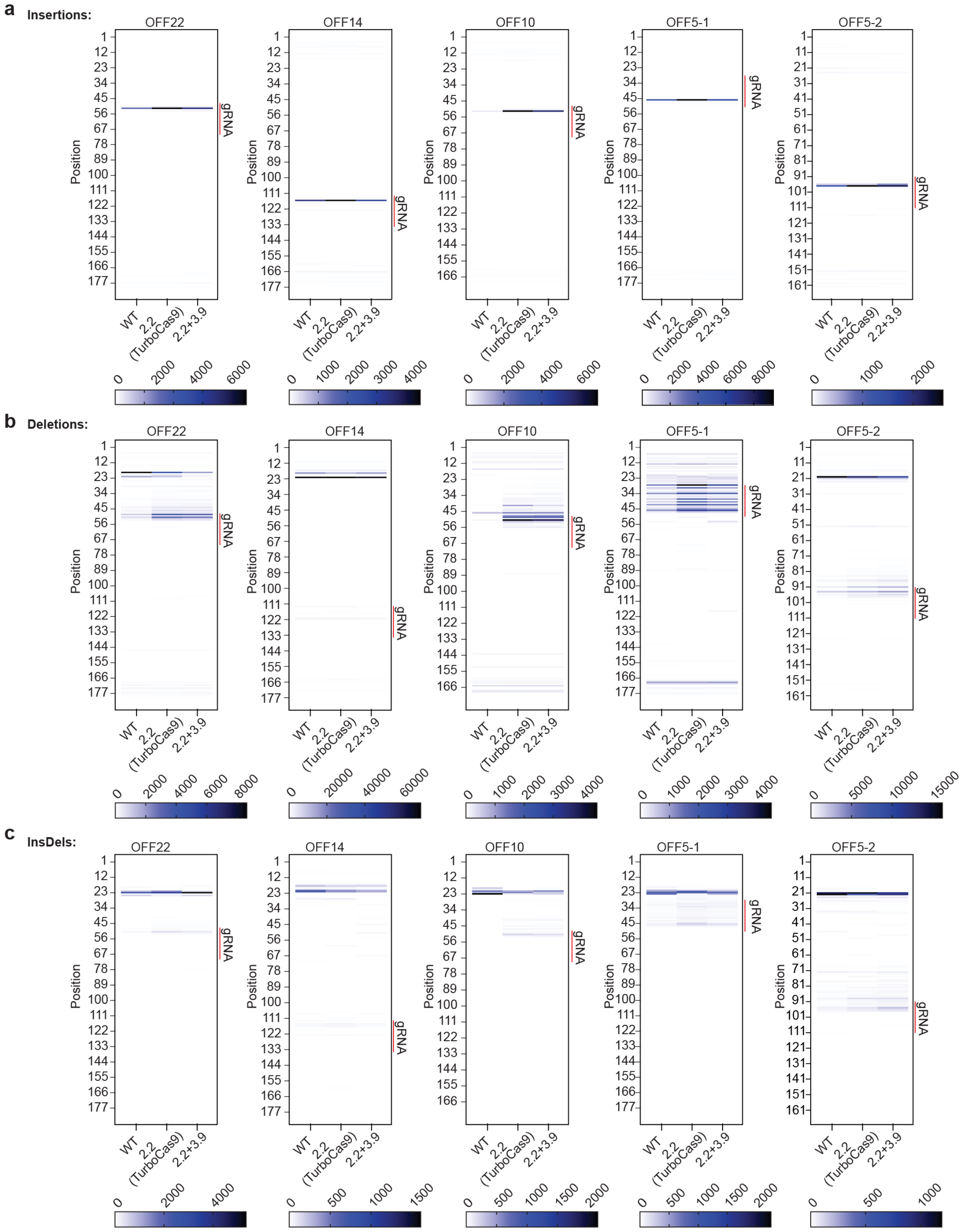

Supplementary Fig. 8 Positional characteristics of off-target mutations. (**a-c**) Heatmaps summarizing the positions of mutations within the top 5 off-target sites for the *VEGFA* gRNA by engineered Cas9 enzymes compared to wild-type (WT) Cas9 in in sequencing data from HEK293T cells. Positional data for the three general classes of editing events: insertions (**a**), deletions (**b**), and combined insertion-deletion events (**c**). Scales bar depict read counts. Location of the gRNA binding sites are shown as red bars.

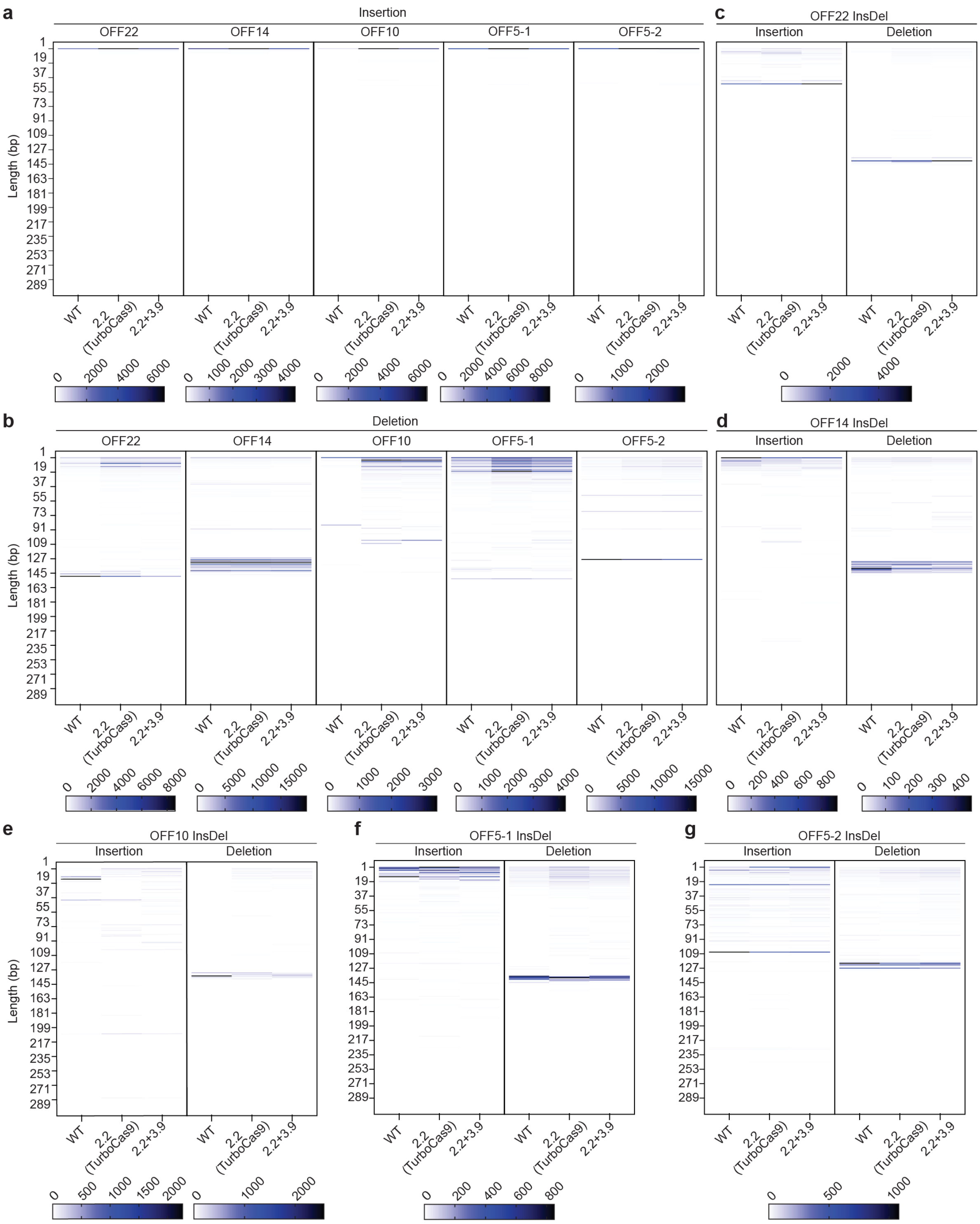

Supplementary Fig. 9 Length distribution of off-target mutations. (**a-c**) Heatmaps summarizing the lengths of mutations in sequencing data from the top 5 off-target sites for the *VEGFA* gRNA by engineered Cas9 enzymes compared to wild-type (WT) Cas9. Length data are summarized for the three general classes of editing events: insertions (**a**), deletions (**b**), and combined insertion-deletion events (**c**). Scale bars depict read counts.

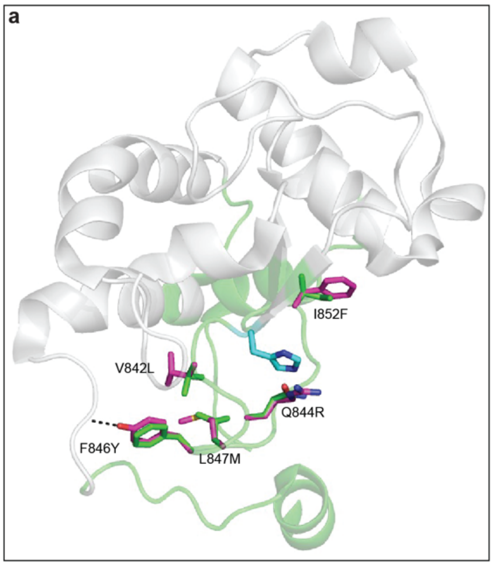

b

Position	WT identity	WT identity occurence in MSA (%)	TurboCas9 identity	TurboCas9 identity occurence in MSA (%)
842	V	15	L	9
844	Q	80	R	16
846	F	82	Y	8
847	L	9	M	1
852	I	54	F	7

Supplementary Fig. 10 Structural and sequence features of TurboCas9 compared to wild-type SpCas9. (**a**) Structural impact of the mutations in TurboCas9 design. Shown is the catalytic domain of Cas9 (PDB ID 5F9R, https://www.wwpdb.org/pdb?id=pdb_00005f9r), with the three mutated regions colored in green. The mutated residues are shown as sticks (wild-type identities in green, designed identities in magenta), and the catalytic His840 is shown in cyan sticks. Mutations V842L, L847M and I852F optimize the core packing, mutation F846Y introduces a new hydrogen bond to the protein backbone carbonyl, and mutation Q844R improves the solvation by introducing a charged residue on the protein surface. (**b**) Frequencies of specific amino acids at the mutated positions of TurboCas9 compared to wild-type SpCas9 in the explored multiple sequence alignment (MSA).
